# Ranking age-specific modifiable risk factors for cardiovascular disease and mortality: evidence from a population-based longitudinal study

**DOI:** 10.1016/j.eclinm.2023.102230

**Published:** 2023-09-27

**Authors:** Fei Tian, Lan Chen, Zhengmin (Min) Qian, Hui Xia, Zilong Zhang, Jingyi Zhang, Chongjian Wang, Michael G. Vaughn, Maya Tabet, Hualiang Lin

**Affiliations:** a Department of Epidemiology, School of Public Health, Sun Yat-sen University, Guangzhou, 510080, China; b Department of Epidemiology and Biostatistics, College for Public Health & Social Justice, Saint Louis University, Saint Louis, MO, 63104, USA; c Center for Health Care, Longhua District, Shenzhen, China; d Department of Epidemiology and Biostatistics, College of Public Health, Zhengzhou University, Zhengzhou, 450001, China; e School of Social Work, Saint Louis University, Saint Louis, MO, 63103, USA; f College of Global Population Health, University of Health Sciences and Pharmacy in St. Louis, 1 Pharmacy Place, St. Louis, MO, 63110, USA

**Keywords:** Cardiovascular disease, Modifiable factor, Age, Epidemiology, Prospective study

## Abstract

**Background:**

Cardiovascular disease (CVD) remains a paramount contemporary health challenge. This study examined age-specific effects of 14 risk factors on CVD and mortality in different age groups.

**Methods:**

We analyzed data from 226,759 CVD-free participants aged 40 years and older in the UK Biobank during the period from baseline time (2006–2010) to September 30, 2021. The primary CVD outcome was a composite of incident coronary artery disease, heart failure, and stroke. We calculated age-specific hazard ratios (HRs) and population-attributable fractions (PAF) for CVD and mortality associated with 14 potentially modifiable risk factors.

**Findings:**

During 12.17-year follow-up, 23,838 incident CVD cases and 11,949 deaths occurred. Age-specific disparities were observed in the risk factors contributing to CVD, and the overall PAF declined with age (PAF of 56.53% in middle-age; 49.78% in quinquagenarian; 42.45% in the elderly). Metabolic factors had the highest PAF in each age group, with hypertension (14.04% of the PAF) and abdominal obesity (9.58% of the PAF) being prominent. Behavioral factors had the highest PAF in the middle-aged group (10.68% of the PAF), and smoking was the leading behavioral factor in all age groups. In socioeconomic and psychosocial risk clusters, low income contributed most among middle-aged (3.74% of the PAF) and elderly groups (3.66% of the PAF), while less education accounted more PAF for quinquagenarian group (4.46% of the PAF). Similar age-specific patterns were observed for cardiovascular subtypes and mortality.

**Interpretation:**

A large fraction of CVD cases and deaths were associated with modifiable risk factors in all age groups. Targeted efforts should focus on the most impactful risk factors, as well as age-specific modifiable risk factors. These findings may inform the development of more precise medical strategies to prevent and manage CVD and related mortality.

**Funding:**

The work was supported by the 10.13039/100000865Bill & Melinda Gates Foundation (grant number: INV-016826 to Hualiang Lin) and the 10.13039/100014717National Natural Science Foundation of China (grant number: 82373534 to Hualiang Lin).

**Research in context**

**Evidence before this study**

Using the search terms (“cardiovascular disease” AND “modifiable risk factor” AND “age” AND “population-based”), we searched PubMed up to July 25, 2023. We retrieved six studies of which only two comprehensively assessed the age-specific associations between modifiable risk factors and cardiovascular disease. However, these two studies focused on a limited selection of modifiable factors and total cardiovascular disease events and mortality, providing limited insights into medical preventive strategies.

**Added value of this study**

CVD risk attributable to modifiable risk factors declined with age Metabolic factors had the highest PAF in each age group, with hypertension and abdominal obesity being prominent. Behavioral factors had the highest PAF in the middle-aged group, and smoking was the leading behavioral factor in all age groups. In socioeconomic and psychosocial risk clusters, low income contributed most in middle-aged and the elderly groups, while less education accounted more PAF for quinquagenarian group.

**Implications of all the available evidence**

The risk of CVD attributable to modifiable factors decreases with age, and there are age-specific variations in the importance of these factors. These results emphasize the need for accurate risk assessment, early detection, and customized interventions at the population level. Additionally, managing individual heart disease risks requires a lifelong and inclusive approach that takes age-specific differences into account.

## Introduction

Cardiovascular disease (CVD), including coronary artery disease (CAD), heart failure, and stroke, remain the leading cause of mortality worldwide. In 2019, it was estimated that 17.7 million years lived with disability (YLDs) and 18.6 million deaths were attributable to CVD.^1^ In the United Kingdom, CVD claimed the lives of 163,000 individuals in 2019, with 73.6% of these cases occurring in the elderly. Furthermore, 1.18 million hospital admissions attributable to CVD were documented, resulting in an immense economic burden of nearly 19 billion pounds.^2^ Due to rising life expectancy, especially in developed countries like the UK, CVD risk is typically associated with advancing age, while morbidity crowd displays a trend of younger average age.^3^

Age is a significant determinant in cardiovascular dysfunction, and its intricate interplay with a plethora of risk factors can have further implications on cardiovascular homeostasis and metabolic disturbances.^4^^,^^5^ Various physiological processes associated with different age groups lead to differing risks of CVD and related health complications across age ranges.^5^ Moreover, age-specific fluctuations in metabolic functions, habitual behaviors, and even socioeconomic conditions might exert disparate impacts on distinct age categories, thereby altering physical susceptibility to CVD. Hence, it is imperative to identify and rank age-specific modifiable risk profiles for devising precise and age-specific preventive policies strategies and management strategies. Although several previous studies have established strong associations of modifiable risk factors with the risk of developing CVD and mortality,^6^^,^^7^ few studies have thus far examined the consistencies or disparities in the relationships between modifiable risk factor profiles when stratified by age. In addition, prior evidence focused on a limited selection of modifiable factors, leaving emerging factors such as sleep, depression, and air pollution out of the framework.^8^ Associations of sleep and depression with CVD is well established.^9^^,^^10^ Air pollution was also considered as an important age-specific risk factor due to the disparities in inflammation responses and physiological vulnerabilities among age groups.^11^ Given this research context, we used data from a nationwide prospective cohort study of UK biobank to unravel and rank the associations and population-attributable fractions (PAF) of 14 potentially modifiable metabolic, behavioral, socioeconomic, psychosocial and air pollution risk factors with CVD and mortality across different age groups.

## Methods

### Study design and participants

UK Biobank is a large-scale, population-based prospective cohort study that aims to enhance the prevention, diagnosis, and treatment of prevalent and life-threatening diseases. Detailed description of the UK biobank cohort has been previously published.^12^ Between 2006 and 2010, over half a million UK residents aged 40–69 years were recruited primarily from urban areas of England, Wales, and Scotland. The baseline measurement was performed at 22 assessment centers throughout the United Kingdom. All participants completed the detailed touch screen health questionnaires and underwent a comprehensive range of physical examinations and biological tests. Of the 502,411 participants who attended baseline assessment, we excluded individuals who were identified at baseline with CVD (N = 20,549) and cancer (N = 42,850). This analysis further excluded those who had self-reported CVD at baseline (N = 13,347). Participants without data on risk factors assessment and other important covariates were also excluded (N = 198,906), leaving 226,759 participants in the final analysis (Figure S1).

### Data collection

Baseline information was collected from 2006 to 2010, and all health-related data on risk factors was gathered by highly trained researchers according to the standardized and detailed research protocols. Most of the data on demographic characteristics, lifestyle behaviors, medical condition, and environmental exposure were measured only once at baseline. Drawing on a priori knowledge and the literature, we identified 14 potentially modifiable risk factors associated with CVD at both individual and population levels.^9^^,^^10^^,^^13^, ^14^, ^15^, ^16^, ^17^, ^18^, ^19^, ^20^, ^21^ These risk factors were grouped into distinct clusters, and detailed definitions and measurement methods for each modifiable risk factor are summarized in Table S1.

Behavioral risk factors included tobacco use, alcohol consumption, diet quality, physical activity, and sleep pattern. Tobacco use was categorized into never, former, or current smokers. Alcohol consumption was evaluated based on reported average standard drinks intake per week of each beverage type. Diet quality was assessed using a diet score based on several food types associated with lower or higher risk of CVD and mortality. The levels of physical activity were evaluated using the self-reported International Physical Activity Questionnaire (IPAQ) and classified as low, moderate, or high.^22^ Sleep quality was evaluated by five chronotypes, duration, insomnia, snoring, and excessive daytime sleepiness.^9^ Metabolic clusters of risk factors consisted of diagnostic hypertension, diabetes, non-HDL cholesterol, and waist-to-hip ratio (an indicator of abdominal obesity). Hypertension was defined as a diagnostic history of hypertension, use of blood pressure-lowering medication, or systolic pressure at baseline ≥140 mmHg or diastolic pressure ≥90 mmHg. Diabetes was defined as a diagnosed diabetes, use of antidiabetic medication, or measured fasting glucose level ≥11.1 mmol/L. Non-HDL cholesterol level referred to total cholesterol value minus HDL cholesterol. Abdominal obesity was identified as a waist-to-hip ratio (WHR) > 0.9 in males and >0.85 in females.^7^

In addition, UK biobank collected data on income, education, and depression to represent socioeconomic and psychological (SEP) factors. Household income was classified as lower than ￡18,000, ￡18,000–30,999, ￡31,000–51,999, ￡52,000–100,000, and higher than ￡100,000. High education was defined as having received higher education or more. Depression was assessed using the Patient Health Questionnaire-2 (PHQ-2), a validated measure that assesses the frequency of depressed mood and anhedonia over the past 2 weeks.^10^ Handgrip strength was evaluated through a JAMAR J00105 hydraulic hand dynamometer, and the average value of both hands was recorded.^19^ Furthermore, ambient PM_2.5_ exposure of each participant was assessed by land-use regression model based on the residential address at baseline. Each modifiable factor was dichotomized and a score of 1 was assigned to indicate an unhealthy status and 0 for a healthy status, as defined in Table S1. The individual scores were then summed to derive the behavioral (0–5), metabolic (0–4), and SEP (0–4) scores, with a higher score indicating a higher risk.^23^^,^^24^

### Follow-up and ascertainment of outcome

All participants were followed-up longitudinally for cardiovascular health outcomes after initial recruitment until censored at the date of first diagnosis of outcomes, death, loss to follow-up, or end of this study (February 28, 2018 for Wales, July 31, 2021 for Scotland, and September 30, 2021 for England), whichever came first. The primary outcomes of interest in our analysis included CVD and individual subtypes (CAD, stroke, and heart failure). Secondary outcomes were cardiovascular mortality and all-cause mortality. These outcomes were captured by linking hospital medical records from Health Episode Statistics in England and Wales and the Scottish Morbidity Records in Scotland and were defined using the International Classification of Diseases 10th revision (ICD-10) (Table S2).

### Statistical analysis

We summarized the characteristics of the participants at baseline by age group and compared characteristic distributions using one-way ANOVA or χ^2^ test, as appropriate.

We applied multivariable Cox proportional hazards models to investigate the associations between modifiable risk factors and cardiovascular outcomes, reporting hazard ratios (HRs) with 95% confidence intervals (CIs). These associations were stratified by age group (middle-aged: <50 years; quinquagenarian: 50 to <60 years; and the elderly: ≥60 years). Meanwhile, we used Schoenfeld residuals method to assess the proportional hazards. In the multivariable-adjusted model, we adjusted for age, sex, ethnicity, region, family history and mutually adjusted for other relevant risk factors. The correlation between these risk factors were examined using Kendall's correlation and found the low level of collinearity (all the coefficients <0.3, Figure S2), and multicollinearity diagnosis indicated no multicollinearity between variables (Table S3). Furthermore, a single product interaction term (age∗each of modifiable risk factor) was incorporated in the model to assess whether the effects of these factors on cardiovascular outcomes varied across different age groups. The *P* values for interaction were calculated using likelihood ratio tests by comparing the models with and without an interaction term. We also assessed the associations between three composite risk scores and incident CVD across age groups. To account for potential multiple comparison issues, false discovery rate with the Benjamini–Hochberg method was used to correct *P* values.

Furthermore, we estimated the average PAF and 95% CIs of cardiovascular events and mortality attributable to individual risk factors following a well-established approach developed by Eide and Gefeller,^25^ and using the ‘averisk’ package in R software developed by Ferguson et al.^26^ In accordance with the procedure of this approach, all the selected modifiable risk factors were dichotomously classified based upon the pre-defined thresholds for these factors. The average PAFs for 14 modifiable risk factors were estimated from a single mutually multivariable-adjusted model for all risk factors. Each risk factor was included in the model in every possible order and the average PAF was calculated by averaging the estimates from all possible permutations. In this approach, the estimated cumulative PAF for different groups of risk factors are additive and total PAFs couldn't exceed 100%. All the PAFs attributable to risk factors should not be less than 0 since it is the lowest threshold to signify risk. The detailed estimation for average PAFs was summarized in Supplementary methods.

### Sensitivity analysis

Several post-hoc sensitivity analyses were conducted. First, we conducted separate age-stratified analyses for men and women to examine whether sex influenced the age-related variations in the associations between risk factors and cardiovascular events and included a product term (age group∗risk factor) in the models to test potential modification effects. Second, we altered the age group threshold to <55 years, 55–<65 years and ≥65 years. Third, we also assigned participants into five age groups (<50 years, 50–<55 years, 55–<60 years, 60–<65 years, and ≥65 years). Fourth, stratum-specific analyses according to follow-up duration were conducted. Fifth, we reran the model with excluding age variable to examine the robustness. Sixth, we impute missing risk factors with multivariate imputation by chained equations and repeat the analyses. Seventh, a sensitivity analysis using Fine & Gray's competing risk models to account for potential competing risk of mortality was performed. Finally, we constructed additive interaction model with two metrics of the relative excess risk due to interaction (RERI) and the attributable proportion due to interaction (AP).

### Ethics statement

Written consent was obtained from every participant prior to recruitment, and the North West Multicenter Research Ethics Committee granted approval for conducting the UK Biobank study (NO.16/NW/0274). We analyzed the data using the UK Biobank Resource under Application Number 69550.

### Role of the funding source

The funders of the study had no role in study design, data collection, data analysis, data interpretation, or writing of the report. FT and HL had full access to all the data in the study and had final responsibility for the decision to submit for publication.

## Results

**Fig. 1:**
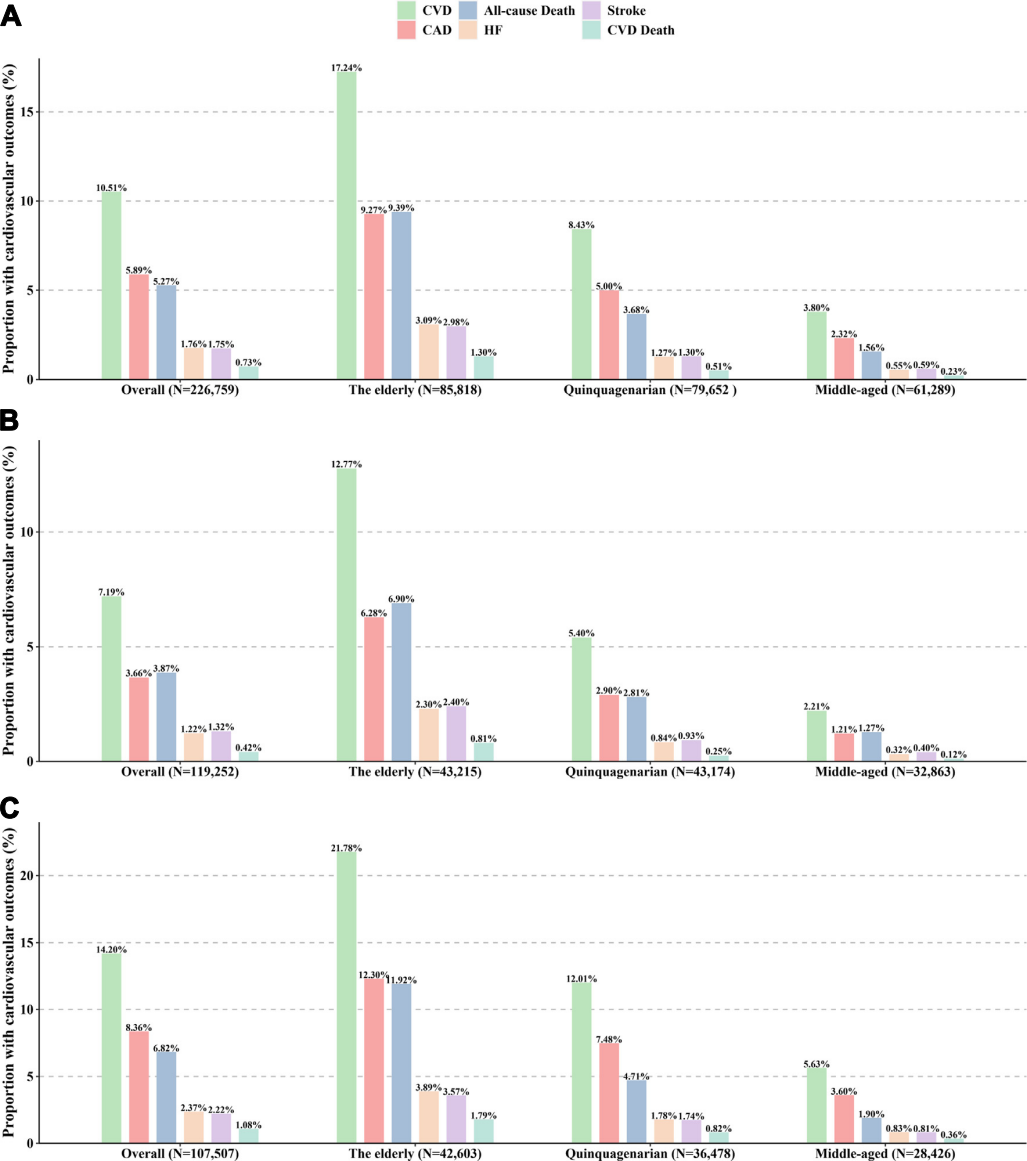
**The proportion of cardiovascular disease events and mortality stratified by age groups in overall population (A), female (B) and male (C).** Abbreviations: CVD, cardiovascular disease; CAD, coronary artery disease; HF, heart failure.

A total of 226,759 participants were enrolled in the analysis. Over a follow-up period of 12.17-years, 23,838 participants developed CVD, with 13,358 from CAD, 3959 from stroke, and 4002 from heart failure. A total of 11,949 all-cause mortality cases were identified, and 13.8% were attributed to CVD. The number of CVD events increased with age, both in the general population and in the male and female populations (Fig. 1). In addition, cardiovascular morbidity was much higher in males than in females, with rates of 14.20% for males and 7.19% for females. The overall CVD event rate was 8.63/1000 person-years, and the CVD incidence rate notably increased with age, ranging from 3.08 for middle-aged individuals to 14.39 for the elderly. The CVD subtypes and mortality event rates were also higher with increasing age. Furthermore, there was a significantly higher CVD incidence in males compared with females (11.72 versus 5.88/1000 person-years) (Figure S3).

### Description of baseline characteristics

At baseline, the mean (±SD) age of all participants was 55.52 (±8.06) years, with 44.91 (±2.75) for middle-aged, 54.69 (±2.88) for quinquagenarian, and 63.88 (±2.81) for the elderly group (Table 1). Of the total cohort, 52.59% were female, and the male-female ratio remained relatively balanced across all age groups. Compared with younger participants, older participants were more likely to be White, current smokers and have poor diet habits, while being less likely to have received higher education, a high income, be depressed, and possess high grip strength. Additionally, older participants were more likely to have diabetes, hypertension, high non-HDL cholesterol levels, and abdominal obesity. The ambient PM_2.5_ exposure concentration was slightly higher in the middle-aged group than the quinquagenarian and elderly groups.

**Table 1:** Baseline characteristics of the study population stratified by age groups.

	Overall	Middle-aged (40 to <50 years)	Quinquagenarian (50 to <60 years)	The elderly (≥60 years)	*P*-value^a^
**No. of participants (%)**	226,759 (100.00)	61,289 (27.03)	79,652 (35.13)	85,818 (37.84)	
**Age, years**	55.52 (8.07)	44.91 (2.75)	54.69 (2.88)	63.88 (2.81)	<0.001
**Sex, n (%)**					<0.001
Male	107,507 (47.41)	28,426 (46.38)	36,478 (45.80)	42,603 (49.64)	
Female	119,252 (52.59)	32,863 (53.62)	43,174 (54.20)	43,215 (50.36)	
**Ethnicity, n (%)**					<0.001
White	216,163 (95.33)	56,415 (92.05)	75,988 (95.40)	83,760 (97.60)	
Nonwhite	10,596 (4.67)	4874 (7.95)	3664 (4.60)	2058 (2.40)	
**Behavioral cluster**					
**Tobacco use, n (%)**					<0.001
Never or former	125,814 (55.48)	37,682 (61.48)	45,060 (56.57)	43,072 (50.19)	
Current	100,945 (44.52)	23,607 (38.52)	34,592 (43.43)	42,746 (49.81)	
**Alcohol use, n (%)**					<0.001
Never or moderate	211,418 (93.23)	57,248 (93.41)	73,715 (92.55)	80,455 (93.75)	
Overdrink	15,341 (6.77)	4041 (6.59)	5937 (7.45)	5363 (6.25)	
**Diet score**					<0.001
<4	124,975 (69.71)	29,776 (62.93)	43,988 (70.14)	51,211 (73.94)	
≥4	54,311 (30.29)	17,538 (37.07)	18,727 (29.86)	18,046 (26.06)	
**Physical activity**					<0.001
Active	159,623 (81.67)	44,052 (81.40)	54,690 (79.63)	60,881 (83.80)	
Inactive	35,820 (18.33)	10,064 (18.60)	13,988 (20.37)	11,768 (16.20)	
**Sleep pattern**					<0.001
Healthy	18,511 (8.16)	5283 (8.62)	6164 (7.74)	7064 (8.23)	
Poor	208,248 (91.84)	56,006 (91.38)	73,488 (92.26)	78,754 (91.77)	
**Metabolic cluster**					
**Hypertension**					<0.001
No	134,706 (59.40)	47,934 (78.21)	49,251 (61.83)	37,521 (43.72)	
Yes	92,053 (40.60)	13,355 (21.79)	30,401 (38.17)	48,297 (56.28)	
**Diabetes**					<0.001
No	204,727 (95.03)	56,590 (97.22)	72,159 (95.41)	75,978 (93.10)	
Yes	10,718 (4.97)	1617 (2.78)	3474 (4.59)	5627 (6.90)	
**Non-HDL cholesterol**					<0.001
Low	75,622 (33.35)	25,517 (41.63)	23,627 (29.66)	26,478 (30.85)	
High	151,137 (66.65)	35,772 (58.37)	56,025 (70.34)	59,340 (69.15)	
**Waist-to-hip ratio**					<0.001
Low	117,561 (51.84)	37,653 (61.44)	41,872 (52.57)	38,036 (44.32)	
High	109,198 (48.16)	23,636 (38.56)	37,780 (47.43)	47,782 (55.68)	
**SEP cluster**					
**Education level**					<0.001
High school or further	115,971 (51.29)	33,557 (54.90)	43,252 (54.41)	39,162 (45.81)	
Less than high school	110,138 (48.71)	27,571 (45.10)	36,235 (45.59)	46,332 (54.19)	
**Household income**					<0.001
High	127,961 (56.54)	44,273 (72.27)	52,332 (65.76)	31,356 (36.69)	
Low	98,344 (43.46)	16,989 (27.73)	27,246 (34.24)	54,109 (63.31)	
**Depression**					<0.001
No	215,000 (94.81)	57,056 (93.09)	75,006 (94.17)	82,938 (96.64)	
Yes	11,759 (5.19)	4233 (6.91)	4646 (5.83)	2880 (3.36)	
**Grip strength**					<0.001
High	133,615 (58.92)	42,181 (68.82)	46,620 (58.53)	44,814 (52.22)	
Low	93,144 (41.08)	19,108 (31.18)	33,032 (41.47)	41,004 (47.78)	
**Air pollution**					
**PM**_**2.5**_**(μg/m**^**3**^**)**	9.96 (1.05)	10.06 (1.07)	9.96 (1.06)	9.89 (1.03)	<0.001

Abbreviations: HDL, high-density lipoprotein; SEP, socioeconomic and psychosocial risk factors; PM_2.5_, fine particulate matter with diameter <2.5 μm.

a *P* values are based on one-way ANOVA for continuous variables or χ^2^ test for categorical variables.

### Association between modifiable risk factors and risk of cardiovascular disease and mortality

**Fig. 2:**
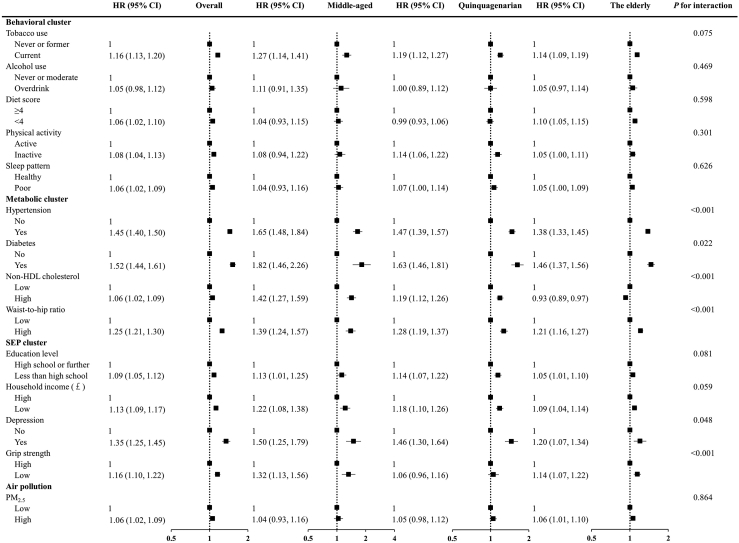
**Associations between 14 modifiable factors and incident cardiovascular disease in overall, middle-aged (38 to <50 years), quinquagenarian (50 to <60 years) and the elderly (≥60 years) groups**. Models were adjusted for age, sex, ethnicity, region, family history, and mutually adjusted for individual risk factors. *P* for interaction was estimated with the use of likelihood ratio test. *P* values were false discovery rate (FDR) corrected. Abbreviations: HR, hazard ratios; HDL, high-density lipoprotein; SEP, socioeconomic and psychosocial risk factors; PM_2.5_, fine particulate matter with diameter <2.5 μm.

**Fig. 3:**
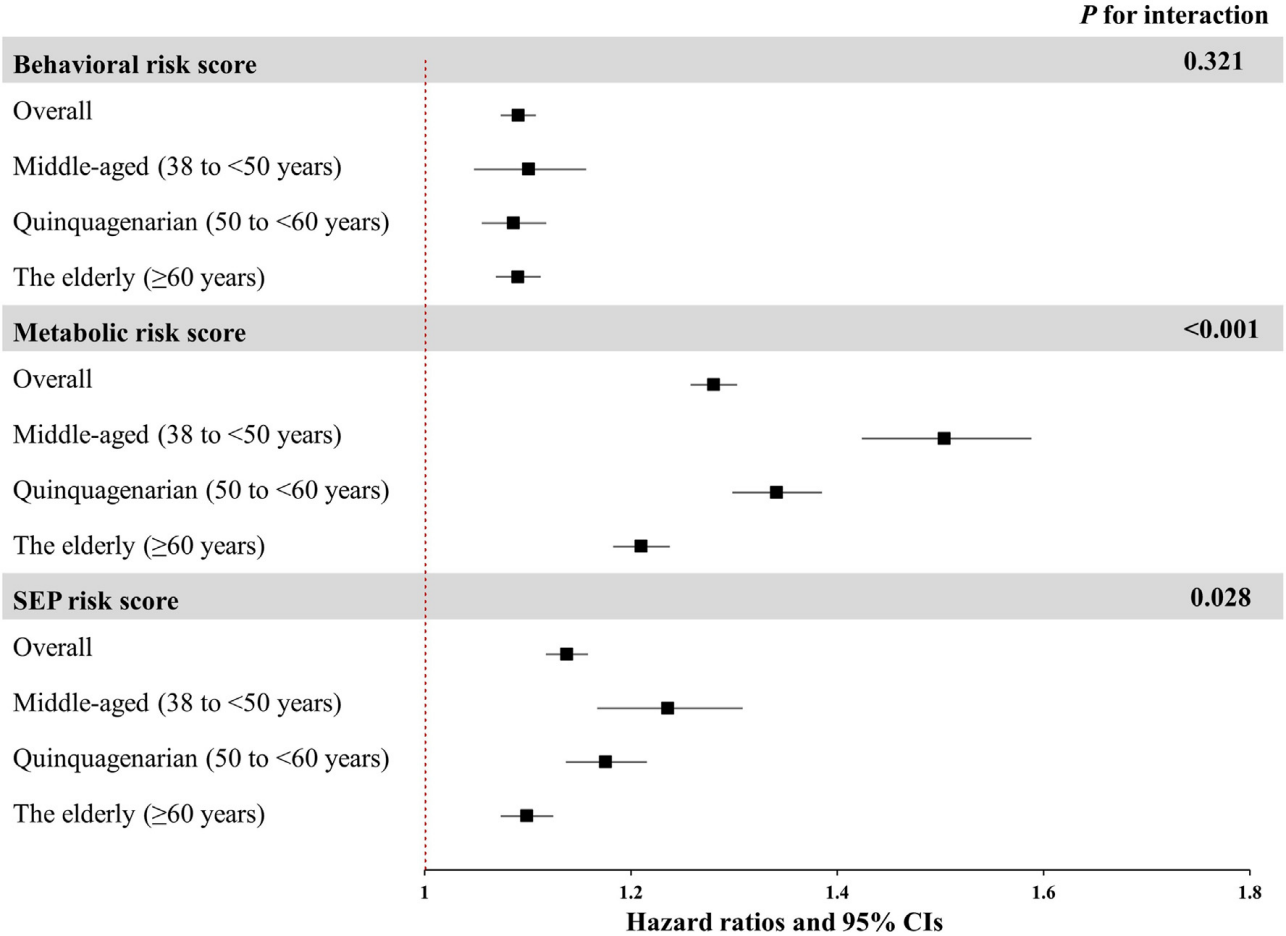
**Hazard ratios and 95% CIs of incident cardiovascular disease associated with each one-point increase in behavioral, metabolic and SEP risk scores by age groups**. Models were adjusted for age, sex, ethnicity, region, family history, and risk scores of other categories. *P* for interaction was estimated with the use of likelihood ratio test. *P* values were false discovery rate (FDR) corrected. Abbreviations: HR, hazard ratios; CIs, confidence intervals; SEP, socioeconomic and psychosocial risk factors.

Fig. 2 shows the associations of each risk factor with risk of incident CVD, stratified by age categories. Generally, the associations between metabolic risk factors and cardiovascular outcomes were stronger than those of socioeconomic and lifestyle risk factors. Specifically, diabetes and hypertension were strongly associated with incident cardiovascular disease across all age groups. The HRs for behavioral risk factors remained similar across age categories. We observed significant interactions of metabolic risk factors with age groups on cardiovascular disease, with the HRs of metabolic risk factors decreasing substantially with age. For instance, the HRs for risk of CVD associated with diabetes were 1.82 (95% CI: 1.46, 2.26) for middle-aged, 1.63 (95% CI: 1.46, 1.81) for quinquagenarian and 1.46 (95% CI: 1.37, 1.56) for the elderly. Concerning socioeconomic and psychological risk factors, we found the estimated HRs related to depressive symptoms and grip strength were stronger and significant for the middle-aged group. Similarly, we observed that the behavioral, metabolic and SEP risk scores were associated with incident CVD among all age groups (Fig. 3), with the HRs decreasing significantly with age for metabolic and SEP score.

The present study also investigated age-specific associations between modifiable risk factors and various cardiovascular outcomes. Similar patterns to age-specific associations of modifiable risk factors were also observed for CAD (Figure S4). Notably, a significant interaction of diet score and physical activity with age groups were additionally found, and lager HRs associated with CAD were observed in the elderly group for low diet quality (HR = 1.13, 95% CI = 1.06, 1.20) and in the quinquagenarian group for physical inactivity (HR = 1.21, 95% CI = 1.11, 1.33). By contrast, for other outcome subtypes, such as stroke and heart failure, as well as cardiovascular mortality, the magnitudes of association of each modified risk factor were similar among the different age groups (Figures S5–S7). For all-cause mortality (Figure S8), we found similar magnitudes of associations for most risk factors among different age groups, except for hypertension, diabetes, and low income. Higher HRs for hypertension (HR = 1.32, 95% CI = 1.20, 1.45) and low income (HR = 1.54, 95% CI = 1.40, 1.71) were found in the quinquagenarian group, and a larger HR for diabetes (HR = 2.29, 95% CI = 1.65, 3.17) was found in the middle-aged group.

The observed associations of modifiable risk factors with CVD were generally consistent for both males and females across age categories (Table S4). However, there was a significant interaction between diabetes and age groups on CVD risk in females which was not observed in males, with stronger effects observed in female quinquagenarian group. In addition, significant interactions between hypertension and age groups on CVD risks were found in both males and females, with stronger effects observed middle-age group (HR = 1.55, 95% CI = 1.36, 1.76 for male; HR = 2.00, 95% CI = 1.61, 2.49). Furthermore, we found that the associations of non-HDL cholesterol level and waist-to-hip ratio with CVD risk decreased with age in both males and females. We also found significant interactions of depression and grip strength with age groups on CVD risk in males and females, respectively.

We reassessed the associations of each risk factor with the risk of incident CVD in sensitivity analyses by altering the age group threshold to include individuals aged <55 years, 55–<65 years, and ≥65 years, and the results of this analysis were consistent with the main findings (Table S5). In addition, the associations were of similar magnitude when assigning participants into five age groups (Table S6). Associations stratified by follow-up duration also yielded the consistent findings (Table S7). Furthermore, when excluding age from the regression model, the effects of some risk factors remained robust, but the unhealthy diet and high PM_2.5_ exposure had potential direction shifts (Table S8). Findings persisted when using multivariate imputation to impute missing data (Table S9) and Fine & Gray's competing risk models (Table S10). Finally, our additive interaction results showed the similar to the findings of multiplicative interaction (Table S11).

### Population attributable fractions for cardiovascular disease and mortality

**Fig. 4:**
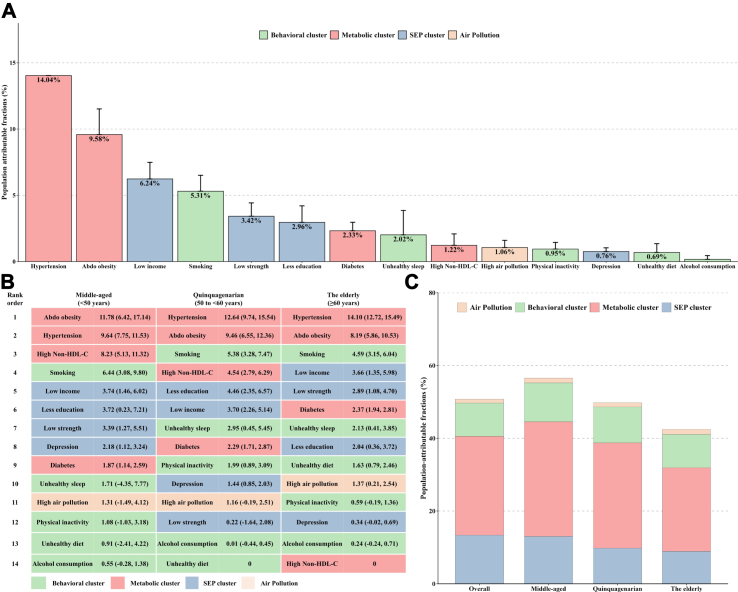
**Population attributable fractions (PAFs) for incident cardiovascular disease associated with the modifiable risk factors. (A) PAFs of each risk factor on cardiovascular disease in overall population; (B) PAFs of each risk factor on cardiovascular disease among age groups; (C) PAFs of risk factor clusters on cardiovascular disease among age groups**. Models were adjusted for age, sex, ethnicity, region, family history, and mutually adjusted for individual risk factors. Estimated PAFs for modifiable risk factors were truncated at a lower limit of 0, as this is the lowest threshold to show a relationship with increased risk. Abbreviations: Abdo obesity, abdominal obesity; non-HDL cholesterol, non-high-density lipoprotein cholesterol; SEP, socioeconomic and psychosocial risk factors.

Overall, the 14 modifiable risk factors identified in this study contributed to more than 50% of PAF for CVD in the general population (Fig. 4A). Hypertension was the largest contributor, accounting for 14.04% of PAF, followed by abdominal obesity (9.58%), low income (6.24%), smoking (5.31%), and low grip strength (3.42%). Other risk factors individually contributed less than 3% of PAF for CVD. Notably, the rankings of risk factors varied when stratified by age (Fig. 4B). Specifically, the proportion of PAF contributed by all modifiable risk factors declined with age (Fig. 4C), with metabolic risk factors reducing remarkably, while hypertension and abdominal obesity remaining the top contributors in all age groups. In the middle-aged group, abdominal obesity, hypertension, and high non-HDL cholesterol were the top three contributors, accounting for 11.78% (95% CI: 6.42%, 17.14%), 9.64% (95% CI: 7.75%, 11.53%), and 8.23% (95% CI: 5.13%, 11.32%) of PAF, respectively. Smoking was consistently the strongest behavioral risk factor across all age groups, with a PAF of 644% (95% CI: 3.08%, 9.80%) in the middle-aged group, 5.38% (95% CI: 3.28%, 7.47%) in the quinquagenarian group, and 4.59% (95% CI: 3.15%, 6.04%) in the elderly group. Other behavioral risk factors jointly contributed less than 5% of the PAF for CVD across all age groups. Socioeconomic and psychological risk factors contributed more in the middle-aged group (Fig. 4C). Low income was the largest socioeconomic and psychological risk factor among middle-aged and the elderly, contributing approximately 3.74% (95% CI: 1.46%, 6.02%) and 3.66% (95% CI: 1.35%, 5.98%) of PAF, respectively, whereas in the quinquagenarian group, low education was the top socioeconomic/psychological risk factor, accounting for 4.46% of the PAF.

In general, the age-specific ranking of modifiable risk factors for CVD remained consistent across different CVD subtypes, namely coronary artery disease, stroke, and heart failure (Figures S9–S11). Hypertension, high non-HDL-cholesterol, and abdominal obesity, consistently occupied the top positions in all age groups, followed by socioeconomic and psychological risk factors, such as low income and low education, and behavioral risk factors, such as smoking. Regarding CAD, abdominal obesity contributed most with a PAF of 14.26% (95% CI: 5.78%, 22.75%) in the middle-aged group and 10.46% (95% CI: 6.94%, 13.97%) in the quinquagenarian group, and hypertension contributed most with a PAF of 15.24% (95% CI: 12.86%, 17.62%) in the elderly group. Hypertension was also the single strongest risk factor for stroke in the overall population and for heart failure in the quinquagenarian and elderly groups. For all-cause mortality (Figure S12), low income had the highest PAF (13.81%) in the overall population, followed by smoking, abdominal obesity, hypertension, and low strength (each contributing to > 6% of the PAF). In the middle-aged group, abdominal obesity contributed most to deaths, but its relative effect was lower in the quinquagenarian and elderly groups (mainly driven by low income and smoking). Similar age-specific variations were also observed for cardiovascular mortality (Figure S13), with metabolic risk factors contributing the most in the overall population and socioeconomic and psychological risk factors contributing increasingly in the quinquagenarian and elderly groups, mainly driven by low income.

## Discussion

In this nationwide prospective cohort of more than 200,000 UK participants, we identified age-specific variations in associations of modifiable risk factors with CVD as well as disparities in magnitudes of corresponding PAFs across age groups. These age-specific disparities provide valuable insights into identification of individuals who are at high risk of CVD, promoting more precise medical strategies, and implementing tailored primary prevention programs that are targeted towards modifiable risk factors.

The overall findings indicate that a significant portion, over 50%, of CVD cases can be attributed to the selected modifiable risk factors. Among these risk factors, metabolic factors were found to have the largest impact, with hypertension being the primary contributor, accounting for more than a fourth of the PAF for CVD. Our findings were in accordance with those from the PURE study, which also reported that hypertension contributed most for the PAF of CVD.^7^^,^^27^, ^28^, ^29^ Our findings that the largest PAFs for death were from low income, contributing about 15% of the population-level risk were inconsistent with prior observations from PURE sub-studies from South America and South Asia, where tobacco use and low education were the largest population-level risks associated with all-cause mortality, respectively.^28^^,^^29^ Both regional variations in risk factor prevalence and distribution and differences in study design, methodology, and population characteristics may contribute to the observed discrepancies.

The age-specific associations between modifiable risk factors and CVD decreased with increasing age, especially for the metabolic cluster of risk factors. In current analysis, despite different degrees of overlap for the CIs of HRs among different age groups, the HRs for metabolic risk factors showed a consistent trend of decreasing with age. When we used more comprehensive scores to examine the association between metabolic factor and CVD, nonoverlap 95% CIs and statistically significant differences were both observed, indicating a significant decline in the magnitudes of the association with advancing age. Consistent with our findings, a cohort study including 1.25 million patients from the UK revealed that the relative risk of systolic or diastolic blood pressure on nearly all CVD (angina, myocardial infarction, heart failure, stroke, cardiac arrest, and peripheral arterial disease) decreased with age.^30^ Similarly, we found that the overall risks of CVD attributable to modifiable risk factors declined noticeably with age. As individuals age, the cumulative effects of modifiable risk factors may become less pronounced due to the additional burden imposed by age-related changes (e.g., arterial stiffness, inflammation, organ function). In other words, age may interact with modifiable risk factors, diminishing their relative contribution to the overall risk. This interaction could explain the declining effect sizes observed in older age groups. Nevertheless, improvements in these modifiable determinants for CVD are still of considerable importance due to that they are the exponentially high absolute risk of CVD for older adults.

Metabolic risk factors accounted for the predominant proportion of PAFs for CVD across age groups, with hypertension, abdominal obesity, and higher non-HDL-cholesterol being the leading risk factors. Our findings emphasized the significance of early detection and effective management of hypertension, as it consistently emerges as a leading risk factor across all age groups. Timely diagnosis, regular blood pressure monitoring, and appropriate pharmacological and lifestyle interventions can help control hypertension and reduce the risk of CVD. Weight management also played a critical role in alleviating the burden of CVD across all age populations. By adopting healthy weight loss strategies, such as dietary modifications and promoting physical activity, about 10% of CVD events may be potentially prevented according to our findings. We also found heterogeneity in the effect of high non-HDL-cholesterol level on overall CVD among different groups, with a high contribution to the PAF among participants aged 60 years and younger, whereas no contributions (even protective effects) were observed for the elderly group. This finding could be partially explained by the component heterogeneity of the subtypes of CVD health outcome within age groups. In line with our observation, a prior study also reported that the magnitude of association of elevated non-HDL-cholesterol level with CAD was stronger compared with other subtypes of CVD, such as stroke and heart failure. In our analysis, a relatively low proportion of CAD was observed in the elderly group, possibly leading to little contribution of non-HDL-cholesterol to the PAF.^7^ In addition, the clinical benefit of LDL-C lowering in older patients remained a topic of debate due to the limited evidence on the elderly in randomized controlled trials.^31^

Our analysis revealed that the risks of CVD associated with behavioral and socioeconomic risk factors exhibited slight variations across different age groups. Notably, in the elderly group, low income emerged as the largest contributor to CVD risk, following high blood pressure and abdominal obesity. A previous review demonstrated that income level was consistently associated with increasing risk of CVD at both individual and regional levels.^32^ Similarly, a cohort study of >40,000 individuals found that income-related inequalities increased CVD risk, especially among the elderly, who had particularly marked association.^33^ Furthermore, our findings highlight the significance of addressing depression as a potential strategy for reducing cardiovascular burden.^10^ The proposed underlying mechanisms linking depression to CVD risk include alterations in autonomic nervous system activity, elevation of catecholamine and inflammatory activity, and endothelial and platelet dysfunction.^34^ In clinical practice, the treatment of depression is primarily aimed at improving mental health and overall well-being. While the direct impact on cardiovascular outcomes requires further investigation, treating depression has been shown to improve quality of life, functional status, and adherence to medical therapies, which would indirectly contribute to better cardiovascular health outcomes.

The contribution of behavioral risk factors to the burden of CVD was primarily driven by smoking, and these effects were broadly consistent for all individuals regardless of age. Smoking is a well-known risk factor for CVD, and while the UK has implemented strict policies to reduce the prevalence of tobacco use over the past three decades resulting in a 30% reduction in total smokers, further efforts to strengthen smoking control could yield significant health benefits for all age groups.^35^ Other lifestyle and socioeconomic risk factors accounted for similar PAFs for CVD among age groups, each contributing to less than 5% of the PAF. Previous analyses have consistently shown the detrimental effects of an unhealthy sleep pattern, unhealthy diet, physical inactivity, excessive drinking, low education, and low grip strength on CVD.^9^^,^^19^^,^^20^^,^^36^, ^37^, ^38^ However, few studies have evaluated the PAF of these lifestyle, behavioral, and socioeconomic determinants on CVD, and even fewer have examined the association of these risk factors with CVD incidence from an age-specific perspective.

In the current analysis, we observed that modifiable risk factors were associated with both cardiovascular and all-cause mortalities, and that the corresponding HRs remained similar across age groups, except for hypertension and diabetes, which had age-specific effects on all-cause mortality. Specifically, we observed an age-specific decline in the risk of all-cause mortality attributable to hypertension and diabetes, with the middle-age group facing a higher risk. In addition, metabolic risk factors still contributed more of the PAF for cardiovascular death among each age group, suggesting that a substantial reduction in CVD death burden could be achieved by hypertension and diabetes control, and management of obesity and weight. Our study findings underscore the substantial contribution of socioeconomic risk factors to the PAF for all-cause death, particularly among the elderly group, accounting for 20% of the PAF. This highlights the critical role of socioeconomic factors in determining health outcomes and mortality rates, which was in line with the evidence in mounting observational studies.^20^^,^^32^^,^^39^ It may be crucial to prioritize measures aimed at improving socioeconomic status (e.g., income and education) and ensuring accessibility to healthcare and social care services, particularly for the elderly population. Our findings also emphasized the contribution of abdominal obesity to all-cause and cardiovascular death in middle-aged individuals, underscoring the need for primary prevention and clinical management of abdominal obesity in middle age to promote better health outcomes and reduce premature mortality.

Among the selected modifiable risk factors, we recognize that certain factors were individually modifiable and had a direct impact on CVD and mortality. These factors include behavioral factors, medical conditions, and metabolic risk factors, which individuals can actively address through lifestyle changes, treatment, and medication. However, socioeconomic and psychosocial risk factors, as well as ambient pollution, may not be directly modifiable at the individual level. These factors are influenced by broader societal and environmental contexts. Nonetheless, concerted efforts at the community and population levels can lead to positive changes in these areas.

In the current study, we identified significant age-specific variations in modifiable risk factors for CVD and mortality among individuals aged 40 years and older. These findings may have important implications for public health practice and strategies. At the population level, by identifying the predominant risk factors within different age groups, healthcare professionals can prioritize interventions that are most effective for each population segment. This targeted approach allows for more precise risk assessment, early detection, and tailored interventions, ultimately reducing the social burden of CVD. At the individual level, it is crucial to adopt a comprehensive framework that integrates age-specific findings, considering the dynamic nature of risk factors and their interactions. This allows for personalized and targeted interventions throughout the entire lifespan, promoting optimal cardiovascular health.

Our study should be interpreted in the context of several potential limitations. First, data was extracted from the UK Biobank cohort, which included only individuals aged 40–69 years old, and thus, the generalizability of our findings to wider age groups may be limited. There is a need for further research specifically targeting the modifiable risk factors and outcomes of premature CVD in younger populations. Second, it should be noted that the PAF estimates presented in our study may not be directly applicable to other populations. PAF is influenced by epidemiological data specific to each country, region, and ethnicity.^40^^,^^41^ Although the risk factors identified in the UK Biobank cohort have demonstrated hazard ratios comparable to those observed in the representative studies, caution should be exercised when generalizing our findings to different populations. Validation across diverse populations is necessary to ensure the effectiveness and appropriateness of the strategies. Third, despite a rigorous adjustment for various confounders and mutual adjustment, our findings may still be susceptible to unmeasured confounding. Additionally, the modifiable risk factors were collected and assessed at baseline, and the relevant information may change over time during the follow-up and this misclassification could potentially bias our results towards the null or create artificial attenuation of associations, leading to an underestimation of the finding, though results stratified by years of follow-up yielded consistent findings. Future studies are warranted to consider collecting information on these factors at multiple time points throughout the follow-up period. Fourth, although some misclassification in risk factors assessment may be inevitable due to the nature of being self-reported, most modifiable risk factors were either obtained from or supplemented by objective measurements (e.g., blood pressure, blood glucose, grip strength, and anthropometry) or reported using validated instruments (e.g., physical activity, food frequency and depression questionnaire). In addition, prior research has observed good agreement between self-reported and primary care data of UK biobank, suggesting the reliability of the self-reported data.^42^ Finally, caution should be exercised when interpreting PAFs comparisons among different risk factors. Only when large differences in PAFs were observed could we draw the conclusion that one risk factor was more significant than another. The estimated PAF stratified by age groups might be more prone to errors, especially when the PAF estimates were small.

In conclusion, the relative risk and population attributable risk of modifiable risk factors for CVD declined with age, and there were age-specific variations in ranking modifiable risk factors. These findings highlight the importance of precise risk assessment, early detection, and tailored interventions at the population level. In addition, managing CVD risk factors at the individual level requires a lifelong perspective and an integrated approach that considers age-specific variations.

## Contributors

H.L. and F.T. conceptualized the study, conducted the formal analyses, visualization, and wrote the original drafts of the manuscript. L.C. and Z.J. provided input to modify the study design. Z.Z. and H.L. provided statistical support and supervision. Z.Q., H.X., C.W., V.M., and T.M. reviewed the manuscript. H.L. and F.T. had full access and verified all the data in the study. All authors were involved in interpretation of results and accept responsibility to submit for publication.

## Data sharing statement

All original data discussed in the text of this paper is also included as figure and/or table either in the main text or in the appendix. Additional information can be supplied by the corresponding author upon reasonable request.

## Declaration of interests

The authors have no conflict of interest to declare.
