# Supplementary material for: Ranking age-specific modifiable risk factors for cardiovascular disease and mortality: evidence from a population-based longitudinal study
Source: eClinicalMedicine. 2023 Sep 27;64:102230. doi: 10.1016/j.eclinm.2023.102230 (PMC10626167; doi:10.1016/j.eclinm.2023.102230)
Supplement: Supplementary Information [file mmc1.docx]

**SUPPLEMENTARY APPENDIX: Ranking age-specific modifiable risk factors for cardiovascular disease and mortality: evidence from a population-based longitudinal study**

**Table of contents**

[Supplementary Methods. Calculation of the Average Population Attributable Fractions 4](#_Toc143028452)

[Table S1. Definitions, and measurements of modifiable risk factors 7](#_Toc143028453)

[Table S2. Cardiovascular disease definitions used in the UK Biobank study 11](#_Toc143028454)

[Table S3. Multicollinearity diagnosis among the modifiable risk factors 12](#_Toc143028455)

[Table S4. Sex-stratified interaction of individual modifiable risk factors with age groups on incident cardiovascular diseases 13](#_Toc143028456)

[Table S5. Hazard ratios of incident cardiovascular diseases associated with individual modifiable risk factors by age groups of <55 years, 55 to <65 years, and ≥ 65 years 15](#_Toc143028457)

[Table S6. Hazard ratios of incident cardiovascular diseases associated with individual modifiable risk factors by age groups of <50 years, 50 to <55 years, 55 to <60 years, 60 to <65 years, and ≥65 years 17](#_Toc143028458)

[Table S7. Associations between modifiable risk factors and cardiovascular diseases in overall population stratified by length of follow-up 19](#_Toc143028459)

[Table S8. Associations between 14 modifiable factors and incident cardiovascular disease in overall, middle-aged (38 to <50 years), quinquagenarian (50 to <60 years) and the elderly (≥60 years) groups when excluding age from the regression model 20](#_Toc143028460)

[Table S9. Hazard ratios and 95% CI of incident cardiovascular diseases associated with individual modifiable risk factors by age groups using multiple imputation to impute data 21](#_Toc143028461)

[Table S10. Hazard ratios and 95% CI of incident cardiovascular diseases associated with individual modifiable risk factors by age groups using Fine & Gray’s competing risk model 23](#_Toc143028462)

[Table S11. Additive interaction between age groups and each modifiable risk factors on risk of CVD 25](#_Toc143028463)

[Table S12. Hazard ratios (95% CIs) of incident cardiovascular diseases associated with individual modifiable risk factors among female and male 26](#_Toc143028464)

[Figure S1. Flowchart of the study participants included and excluded in study 28](#_Toc143028465)

[Figure S3. The event rates of cardiovascular disease events and mortality stratified by age groups in overall population (A), female (B) and male (C) 30](#_Toc143028466)

[Figure S4. Associations between 14 modifiable factors and incident coronary artery disease in middle-aged (38 to <50 years), quinquagenarian (50 to <60 years) and the elderly (≥60 years) groups. 31](#_Toc143028467)

[Figure S5. Associations between 14 modifiable factors and incident stroke in middle-aged (38 to <50 years), quinquagenarian (50 to <60 years) and the elderly (≥60 years) groups. 32](#_Toc143028468)

[Figure S6. Associations between 14 modifiable factors and incident heart failure in middle-aged (38 to <50 years), quinquagenarian (50 to <60 years) and the elderly (≥60 years) groups 33](#_Toc143028469)

[Figure S7. Associations between 14 modifiable factors and cardiovascular mortality in middle-aged (38 to <50 years), quinquagenarian (50 to <60 years) and the elderly (≥60 years) groups 34](#_Toc143028470)

[Figure S8. Associations between 14 modifiable factors and all-cause mortality in middle-aged (38 to <50 years), quinquagenarian (50 to <60 years) and the elderly (≥60 years) groups 35](#_Toc143028471)

[Figure S9. Ranking the population attributable fractions for incident coronary artery disease associated with the 14 modifiable risk factors among age groups 36](#_Toc143028472)

[Figure S10. Ranking the population attributable fractions for incident stroke associated with the 14 modifiable risk factors among age groups 37](#_Toc143028473)

[Figure S11. Ranking the population attributable fractions for incident heart failure associated with the 14 modifiable risk factors among age groups. 38](#_Toc143028474)

[Figure S12. Ranking the population attributable fractions for cardiovascular mortality associated with the 14 modifiable risk factors among age groups 39](#_Toc143028475)

[Figure S13. Ranking the population attributable fractions for all-cause mortality associated with the 14 modifiable risk factors among age groups 40](#_Toc143028476)

# Supplementary Methods. Calculation of the Average Population Attributable Fractions

The population attributable fraction is a more relevant metric for this purpose and quantifies the proportional reduction in disease prevalence that would be achieved if the risk factor could be somehow eliminated from the population. The equation of average PAF ($\lambda$) for risk factor *k*, can be represented as ^1^:

$$\lambda_{a,k}=\left\{ \sum_{\sigma\boldsymbol{\in}S_{K}\mathbf{:}\sigma\left( k \right)\mathbf{=}1} \boldsymbol{\lambda}_{k}+ \right.\left. \sum_{\sigma\boldsymbol{\in}S_{K}\mathbf{:}\sigma\left( k \right)\boldsymbol{\neq}1} \boldsymbol{\lambda}_{k|\sigma^{\mathbf{-}1}\mathbf{(}1\boldsymbol{)\ldots}\sigma^{\mathbf{-}1}\mathbf{(}\sigma\mathbf{(}k\mathbf{)-}1\mathbf{)}} \right\}/K!$$

*σ* represent some permutation function over the integers 1, …, K, that is an invertible function σ: {1, …, K} → {1, …, K}. For example, a permutation *σ* with $\sigma(1)=5$ represents a removal order where risk factor ‘1’ is the 5th risk factor to be eliminated from the population. We denote the set of all possible permutation functions by$S_{K}$.While the expression $\boldsymbol{\lambda}_{k|\sigma\mathbf{-}1\mathbf{(}1\boldsymbol{)\ldots}\sigma\mathbf{-}1\mathbf{(}\sigma\mathbf{(}k\mathbf{)-}1\mathbf{)}}$ looks complex, it is simply the sequential attributable fraction from removing risk factor *k* having already removed the risk factors labelled: $\sigma^{\mathbf{-}1}\mathbf{(}1\boldsymbol{)\ldots}\sigma^{\mathbf{-}1}\mathbf{(}\sigma\mathbf{(}k\mathbf{)-}1\mathbf{)}$. Note that when $\sigma(k)\neq1,\sigma-1(1)\ldots\sigma-1(\sigma(k)-1)$ is the set of risk factors that appear before risk factor k, according to the order specified by σ. To make this more concrete, consider a situation with K = 4 risk factors, where we want to calculate the sequential attributable fraction corresponding to removing 2, after having removed first 4, then 3 and then 1, that is: λ_2|4,3,1_. Then, since 4 is the first risk factor to be removed, $\sigma(4)=1$. Similarly, $\sigma(3)=2$, $\sigma(1)=3$ and$\sigma(2)=4$.. Applying the inverse permutation, it follows then that $\sigma^{-1}(1)=1$, $\sigma^{-1}(2)=3$, $\sigma^{-1}(3)=1$, which are the three risk factors that are removed before removing $\sigma^{-1}(4)=2$.

Average population attributable fractions are calculated for a set of risk factors (either binary or ordinal valued), which was designed for prospective studies. Though this calculation based on logistic regression like the other computational methods, it can partition a well-defined combined attributable fraction into individual contributions for each risk factor and avoid calculating cases where the sum is greater than one, and it has been widely applied in the large prospective studies ^2-5^. Confidence intervals are found by Monte Carlo simulation. In addition to an exact calculation of PAF, an approximate calculation, based on randomly sampling permutations has been implemented to ensure the calculation is computationally tractable when the number of risk factors is large.

Average population attributable fractions (APAFs) and their 95% confidence intervals were calculated after mutual adjustments, using the approach described by Eide and Olaf ^6,7^. This approach involves logistic regression, where all risk factors of interest are added to the model in every possible order, and the average of all PAFs are then calculated using the R package called ‘averisk’ developed by Ferguson et. al. Estimates of PAF were further adjusted for age, sex, and ethnicity. This APAF calculation allows for an estimation of the individual contribution of each risk factor (or group of risk factors) to the overall PAF, and generally provides a smaller estimate for PAF for individual risk factors than more conventional methods used by GBD, and in INTEREART and INTERSTROKE, for which the sum of modifiable risk factor PAFs exceed the cumulative PAF. The ‘averisk’ package also computes confidence intervals based on Monte Carlo simulation. PAFs for selected modifiable risk factors were calculated together using a single model.

**References:**

1. Ferguson J, Alvarez-Iglesias A, Newell J, Hinde J, O'Donnell M. Estimating average attributable fractions with confidence intervals for cohort and case-control studies. Stat Methods Med Res 2018; 27(4): 1141-52.

2. Yusuf S, Joseph P, Rangarajan S, et al. Modifiable risk factors, cardiovascular disease, and mortality in 155 722 individuals from 21 high-income, middle-income, and low-income countries (PURE): a prospective cohort study. Lancet 2020; 395(10226): 795-808.

3. Eide GE, Gefeller O. Sequential and average attributable fractions as aids in the selection of preventive strategies. Journal of Clinical Epidemiology 1995; 48(5): 645-55.

4. Yusuf S, Joseph P, Rangarajan S, et al. Modifiable risk factors, cardiovascular disease, and mortality in 155 722 individuals from 21 high-income, middle-income, and low-income countries (PURE): a prospective cohort study. Lancet 2020; 395(10226): 795-808.

5. Walli-Attaei M, Rosengren A, Rangarajan S, et al. Metabolic, behavioural, and psychosocial risk factors and cardiovascular disease in women compared with men in 21 high-income, middle-income, and low-income countries: an analysis of the PURE study. The Lancet 2022; 400(10355): 811-21.

6. Li S, Liu Z, Joseph P, et al. Modifiable risk factors associated with cardiovascular disease and mortality in China: a PURE substudy. European Heart Journal 2022; 43(30): 2852-2863.

7. Lopez-Jaramillo P, Joseph P, Lopez-Lopez JP, et al. Risk factors, cardiovascular disease, and mortality in South America: a PURE substudy. European Heart Journal 2022; 43(30): 2841-51.

# Table S1. Definitions, and measurements of modifiable risk factors

| **Risk Factor** | **Definition or method of**  **measurement** | **Risk category used for calculation of PAF** | **Reference category used for calculation of PAF** |
| --- | --- | --- | --- |
| **Behavioral cluster of risk factors:** | | | |
| **Tobacco use** | Self-reported current/past smoking status, categorized as  never, former, or current. | History of current or former tobacco use | No history of tobacco use |
| **Alcohol** ^1^ | Self-reported average standard drinks consumed  per week of each beverage type. | Binge drinking as consumption of ≥ 6  standard drinks/day for women or ≥ 8 standard drinks/day for men. No drinking was also regarding as risk group. | Moderate drinking as consumption of < 6 standard drinks/day for women or < 8 standard drinks/day for men. |
| **Diet** | Diet was measured via the Oxford WebQ, a web-based 24h recall. Diet score was created by several food types associated with a lower or higher risk of CVD and mortality: fruits, vegetables, fish, whole grain (for which the higher, the better), red meat, processed product, and refined grain (for which the lower, the better). We computed this score, which ranged from 0 (poor) to 7 (healthy). | Diet score < 4 | Diet score ≥ 4 |
| **Physical**  **activity** | Physical activity, the levels were evaluated by adopting the self-reported International Physical Activity Questionnaire (IPAQ), which included 3 types of activity (walking, moderate, and vigorous intensity activities). Regular activity was described as≥150 minutes moderate activity per week or ≥ 75 minutes vigorous activity per week or equivalent combination or moderate physical activity at least 5 days a week or vigorous activity once a week | Definition of regular activity was not met | Definition of regular activity was met |
| **Sleep pattern ^2^** | Five sleep factors (chronotype, duration, insomnia, snoring,  and excessive daytime sleepiness) were used to generate a healthy sleep score. Early chronotype (‘morning’ or ‘morning than evening’); sleep 7–8 h per day; reported never or rarely insomnia symptoms; no self-reported snoring; and no frequent daytime sleepiness (‘never/rarely’ or ‘sometimes’) were considered as low risk and got one score. All component scores were summed to obtain a healthy sleep score ranging from 0 to 5, with higher scores indicating a healthier sleep pattern. | Sleep score < 4 | Sleep score ≥ 4 |
| **Metabolic cluster of risk factors:** | | | |
| **Hypertension** | Hypertension was defined as meeting at least one of three criteria: (1)  self-reported doctor diagnosis of hypertension, (2) self-reported use of antihypertensive medication, or (3) measured systolic blood pressure ≥140 mm of mercury (mmHg) or diastolic blood pressure ≥90 mmHg. | Definition of hypertension was met | Definition of hypertension was not met |
| **Diabetes** | Diabetes was defined as meeting at least one of three criteria: (1)  self-reported doctor diagnosis of diabetes, (2) self-reported use of hypoglycemic medication, or (3) measured random glucose level ≥11.1 mmol/L or glycated  hemoglobin (HbA1c) level ≥48 mmol/mol (6.5%). | Definition of diabetes was met | Definition of diabetes was not met |
| **Abdominal**  **obesity** | Waist and hip circumference were measured routinely in participants  at baseline, and used to calculate  the waist to hip ratio (WHR) | WHR > 0.9 in men or 0.85 in women | WHR < 0.9 in men or 0.85 in women |
| **Non-HDL cholesterol** | Total cholesterol minus HDL, measured using fasting lipid values | Highest tertile of non-HDL cholesterol | Lowest two tertiles of non-HDL cholesterol |
| **Socio-economic and psychosocial cluster of risk factors:** | | | |
| **Education level** | Self-reported education status, and classified as high (college education) and low (any school degree, vocational qualifications, and other) | Low education | High education |
| **Household income** | Self-reported before-tax household income, and classified as less than ￡18,000, ￡18,000–30,999, ￡31,000–51,999, ￡50,000–100,000, and higher than ￡100,000. | Household income < ￡30,999 | Household income ≥ ￡30,999 |
| **Depression ^3^** | Using the Patient Health Questionnaire-2 (PHQ-2) with 2 items asking the frequency of depressed mood and anhedonia over the past 2 weeks, with response options being “not at all,” “several days,” “more than half the days,” and “nearly every day,” scored as 0, 1, 2, and 3, respectively. Thus, the PHQ-2 score can range from 0 to 6; a score 3 of more is indicative of possible depressive disorder. | Depression score > 3 | Depression score ≤ 3 |
| **Grip strength ^4^** | Handgrip strength was evaluated through a JAMAR J00105 hydraulic hand dynamometer and the average value of both hands. | Lowest two quintiles of grip strength | Highest three quintiles of grip strength |
| **Ambient air pollution** | | | |
| **PM_2.5_** | A standardized land use regression model was used to calculate the annual average concentrations of PM_2.5_. | Highest tertile of PM_2.5_ concentration | Lowest two tertiles of PM_2.5_ concentration |
| Abbreviations: PAF, population attributable fraction; HDL, high-density lipoprotein; WHR, waist to hip ratio; PM_2.5_, fine particulate matter with diameter <2.5 μm.  **References:**  1. Tu SJ, Gallagher C, Elliott AD, et al. Risk Thresholds for Total and Beverage-Specific Alcohol Consumption and Incident Atrial Fibrillation. JACC Clin Electrophysiol 2021; 7(12): 1561-9.  2. Fan M, Sun D, Zhou T, et al. Sleep patterns, genetic susceptibility, and incident cardiovascular disease: a prospective study of 385 292 UK biobank participants. Eur Heart J 2020; 41(11): 1182-9.  3. Harshfield EL, Pennells L, Schwartz JE, et al. Association Between Depressive Symptoms and Incident Cardiovascular Diseases. Jama 2020; 324(23): 2396-405.  4. Yates T, Zaccardi F, Dhalwani NN, et al. Association of walking pace and handgrip strength with all-cause, cardiovascular, and cancer mortality: a UK Biobank observational study. Eur Heart J 2017; 38(43): 3232-40. | | | |

# Table S2. Cardiovascular disease definitions used in the UK Biobank study

| **Diseases** | **ICD-9** | **ICD-10** | **Self-reported field** |
| --- | --- | --- | --- |
| **Coronary artery disease** | 410, 4109, 411, 4119, 412, 4129,  4140, 4148, 4149 | I21, I21.0, I21.1, I21.2, I21.3, I21.4, I21.9, I22, I22.0, I22.1, I22.8, I22.9, I23, I23.0, I23.1, I23.2, I23.3, I23.4, I23.5, I23.6, I23.8, I24, I24.0, I24.1, I24.8, I24.9, I25.1, I25.2, I25.5, I25.6, I25.8, I25.9 | 6150(1), 3894, 20004(1070,  1095, 1523) |
| **Heart failure** | 4254, 4280, 4281, 4289 | I11.0 I11.3, I13.2, I25.5, I42.0, I42.1, I42.2, I42.5, I42.8, I42.9, I50, I50.1, I50.9 | 20002(1076) |
| **Stroke** | 430X^*^, 431X, 434X, 4340, 4341, 4349, 464X | I60, I60.X, I61, I61.0, I61.1, I61.2, I61.3, I61.4, I61.5, I61.6, I61.8, I61.9, I63, I63.0, I63.1, I63.2, I63.3, I63.4, I63.5, I63.6, I63.8, I63.9, I64.X | 6150(3), 4056, 20002(1081,  1491, 1583, 1086) |

^*^An ICD code suffixed with ‘X’ stands for any code starting with the figures preceding the X

# Table S3. Multicollinearity diagnosis among the modifiable risk factors

| **Risk factors** | **Multicollinearity diagnostics** | | |
| --- | --- | --- | --- |
|  | **Eigen value** | **Variance inflation** | **Tolerance** |
| Smoking | 0.378593 | 1.057304 | 0.9458020 |
| Alcohol consumption | 0.325598 | 1.029362 | 0.9714754 |
| Unhealthy diet | 0.242357 | 1.073487 | 0.9315435 |
| Physical inactivity | 0.232622 | 1.023389 | 0.9771457 |
| Unhealthy sleep | 0.227973 | 1.014832 | 0.9853847 |
| Hypertension | 0.216312 | 1.113514 | 0.8980575 |
| Diabetes | 0.212952 | 1.090257 | 0.9172147 |
| Non-HDL cholesterol | 0.181263 | 1.039218 | 0.9622616 |
| Obesity | 0.172845 | 1.259590 | 0.7939094 |
| Less education | 0.164708 | 1.111772 | 0.8994651 |
| Low household income | 0.137639 | 1.254263 | 0.7972807 |
| Depression | 0.053154 | 1.030627 | 0.9702831 |
| Low grip strength | 0.044406 | 2.195456 | 0.4554862 |
| High air pollution | 0.041777 | 1.019185 | 0.9811764 |

Abbreviations: HDL, high-density lipoprotein.

# Table S4. Sex-stratified interaction of individual modifiable risk factors with age groups on incident cardiovascular diseases

| **Modifiable factors** | **HR (95% CI)** | | | ***P* for interaction^*^** |
| --- | --- | --- | --- | --- |
|  | **Middle-aged** | **Quinquagenarian** | **The elderly** |  |
| **Behavioral cluster** |  |  |  |  |
| **Tobacco use** |  |  |  |  |
| Never or former | 1 | 1 | 1 |  |
| Current (Male) | 1.23 (1.09, 1.40) | 1.18 (1.10, 1.28) | 1.12 (1.06, 1.18) | 0.117 |
| Current (Female) | 1.38 (1.13, 1.68) | 1.23 (1.10, 1.37) | 1.18 (1.10, 1.27) | 0.473 |
| **Alcohol use** |  |  |  |  |
| Never or moderate | 1 | 1 | 1 |  |
| Overdrink (Male) | 1.16 (0.94, 1.44) | 1.04 (0.91, 1.18) | 1.09 (0.99, 1.19) | 0.314 |
| Overdrink (Female) | 0.89 (0.57, 1.41) | 0.89 (0.69, 1.14) | 0.93 (0.77, 1.13) | 0.912 |
| **Diet score** |  |  |  |  |
| ≥4 | 1 | 1 | 1 |  |
| <4 (Male) | 1.09 (0.97, 1.24) | 0.98 (0.91, 1.06) | 1.05 (1.00, 1.11) | 0.253 |
| <4 (Female) | 0.89 (0.72, 1.10) | 1.02 (0.90, 1.16) | 1.22 (1.12, 1.33) | 0.337 |
| **Physical activity** |  |  |  |  |
| Active | 1 | 1 | 1 |  |
| Inactive (Male) | 1.06 (0.91, 1.24) | 1.08 (0.99, 1.18) | 1.06 (0.99, 1.14) | 0.537 |
| Inactive (Female) | 1.10 (0.87, 1.40) | 1.24 (1.09, 1.40) | 1.04 (0.94, 1.14) | 0.145 |
| **Sleep pattern** |  |  |  |  |
| Healthy | 1 | 1 | 1 |  |
| Poor (Male) | 1.07 (0.93, 1.22) | 1.04 (0.97, 1.13) | 1.07 (1.01, 1.13) | 0.523 |
| Poor (Female) | 0.98 (0.80, 1.20) | 1.12 (1.00, 1.25) | 1.01 (0.94, 1.08) | 0.367 |
| **Metabolic cluster** |  |  |  |  |
| **Hypertension** |  |  |  |  |
| No | 1 | 1 | 1 |  |
| Yes (Male) | 1.55 (1.36, 1.76) | 1.40 (1.30, 1.51) | 1.35 (1.28, 1.43) | 0.033 |
| Yes (Female) | 2.00 (1.61, 2.49) | 1.63 (1.46, 1.82) | 1.43 (1.33, 1.54) | 0.002 |
| **Diabetes** |  |  |  |  |
| No | 1 | 1 | 1 |  |
| Yes (Male) | 1.86 (1.44, 2.40) | 1.50 (1.32, 1.71) | 1.42 (1.31, 1.53) | 0.146 |
| Yes (Female) | 1.69 (1.12, 2.56) | 2.02 (1.65, 2.47) | 1.60 (1.41, 1.82) | 0.033 |
| **Non-HDL cholesterol** |  |  |  |  |
| Low | 1 | 1 | 1 |  |
| High (Male) | 1.44 (1.27, 1.63) | 1.23 (1.14, 1.33) | 0.91 (0.86, 0.97) | <0.001 |
| High (Female) | 1.33 (1.06, 1.67) | 1.08 (0.97, 1.21) | 0.95 (0.88, 1.02) | 0.004 |
| **Waist-to-hip ratio** |  |  |  |  |
| Low | 1 | 1 | 1 |  |
| High (Male) | 1.27 (1.11, 1.45) | 1.23 (1.13, 1.35) | 1.19 (1.12, 1.27) | 0.043 |
| High (Female) | 1.72 (1.40, 2.13) | 1.31 (1.17, 1.47) | 1.22 (1.14, 1.31) | <0.001 |
| **SEP cluster** |  |  |  |  |
| **Education level** |  |  |  |  |
| High school or further | 1 | 1 | 1 |  |
| Less than high school (Male) | 1.10 (0.97, 1.25) | 1.13 (1.05, 1.22) | 1.07 (1.01, 1.13) | 0.170 |
| Less than high school (Female) | 1.19 (0.97, 1.46) | 1.17 (1.05, 1.30) | 1.03 (0.96, 1.11) | 0.035 |
| **Household income (￡)** |  |  |  |  |
| High | 1 | 1 | 1 |  |
| Low (Male) | 1.11 (0.95, 1.28) | 1.17 (1.08, 1.27) | 1.10 (1.04, 1.16) | 0.173 |
| Low (Female) | 1.47 (1.19, 1.80) | 1.17 (1.04, 1.31) | 1.07 (0.98, 1.16) | 0.003 |
| **Depression** |  |  |  |  |
| No | 1 | 1 | 1 |  |
| Yes (Male) | 1.42 (1.13, 1.78) | 1.47 (1.27, 1.71) | 1.08 (0.93, 1.26) | 0.007 |
| Yes (Female) | 1.62 (1.21, 2.18) | 1.40 (1.16, 1.70) | 1.38 (1.16, 1.63) | 0.341 |
| **Grip strength** |  |  |  |  |
| High | 1 | 1 | 1 |  |
| Low (Male) | 1.10 (0.81, 1.51) | 1.16 (0.99, 1.35) | 1.14 (1.05, 1.24) | 0.592 |
| Low (Female) | 1.46 (1.19, 1.78) | 0.99 (0.88, 1.12) | 1.15 (1.04, 1.28) | 0.021 |
| **Air pollution** |  |  |  |  |
| **PM_2.5_** |  |  |  |  |
| Low | 1 | 1 | 1 |  |
| High (Male) | 1.11 (0.98, 1.26) | 1.02 (0.94, 1.10) | 1.03 (0.97, 1.08) | 0.383 |
| High (Female) | 0.89 (0.73, 1.09) | 1.10 (0.99, 1.23) | 1.11 (1.03, 1.20) | 0.531 |

Abbreviations: HR, hazard ratios; HDL, high-density lipoprotein; SEP, socioeconomic and psychosocial risk factors; PM_2.5_, fine particulate matter with diameter <2.5 μm. Models were adjusted for ethnicity and mutually adjusted for individual risk factors.

**^*^** *P* for interaction was estimated with the use of likelihood ratio test by comparing two models with and without interaction term (age* risk factor) within each sex group. *P* values were false discovery rate (FDR) corrected.

# Table S5. Hazard ratios of incident cardiovascular diseases associated with individual modifiable risk factors by age groups of <55 years, 55 to <65 years, and ≥ 65 years

| **Modifiable factors** | **HR (95% CI)** | | | ***P* for interaction** |
| --- | --- | --- | --- | --- |
|  | **<55 years** | **55 to <65 years** | **≥65 years** |  |
| **Behavioral cluster** |  |  |  |  |
| **Tobacco use** |  |  |  | <0.001 |
| Never or former | 1 | 1 | 1 |  |
| Current | 1.22 (1.13, 1.31) | 1.18 (1.13, 1.24) | 1.11 (1.05, 1.18) |  |
| **Alcohol use** |  |  |  | 0.379 |
| Never or moderate | 1 | 1 | 1 |  |
| Overdrink | 1.05 (0.92, 1.20) | 1.04 (0.96, 1.14) | 1.03 (0.91, 1.17) |  |
| **Diet score** |  |  |  | 0.577 |
| ≥4 | 1 | 1 | 1 |  |
| <4 | 1.00 (0.93, 1.08) | 1.08 (1.03, 1.14) | 1.07 (1.01, 1.15) |  |
| **Physical activity** |  |  |  | 0.171 |
| Active | 1 | 1 | 1 |  |
| Inactive | 1.10 (1.01, 1.20) | 1.08 (1.02, 1.14) | 1.06 (0.98, 1.14) |  |
| **Sleep pattern** |  |  |  | 0.048 |
| Healthy | 1 | 1 | 1 |  |
| Poor | 1.02 (0.95, 1.10) | 1.09 (1.04, 1.15) | 1.00 (0.95, 1.06) |  |
| **Metabolic cluster** |  |  |  |  |
| **Hypertension** |  |  |  | <0.001 |
| No | 1 | 1 | 1 |  |
| Yes | 1.57 (1.46, 1.70) | 1.40 (1.34, 1.47) | 1.38 (1.30, 1.47) |  |
| **Diabetes** |  |  |  | <0.001 |
| No | 1 | 1 | 1 |  |
| Yes | 1.73 (1.50, 1.99) | 1.63 (1.51, 1.76) | 1.32 (1.20, 1.44) |  |
| **Non-HDL cholesterol** |  |  |  | <0.001 |
| Low | 1 | 1 | 1 |  |
| High | 1.33 (1.24, 1.43) | 1.06 (1.01, 1.11) | 0.85 (0.79, 0.90) |  |
| **Waist-to-hip ratio** |  |  |  | <0.001 |
| Low | 1 | 1 | 1 |  |
| High | 1.35 (1.25, 1.47) | 1.22 (1.16, 1.29) | 1.21 (1.13, 1.30) |  |
| **SEP cluster** |  |  |  |  |
| **Education level** |  |  |  | 0.001 |
| High school or further | 1 | 1 | 1 |  |
| Less than high school | 1.13 (1.05, 1.22) | 1.10 (1.05, 1.15) | 1.04 (0.98, 1.11) |  |
| **Household income (￡)** |  |  |  | <0.001 |
| High | 1 | 1 | 1 |  |
| Low | 1.30 (1.20, 1.41) | 1.10 (1.05, 1.16) | 1.06 (0.99, 1.13) |  |
| **Depression** |  |  |  | 0.225 |
| No | 1 | 1 | 1 |  |
| Yes | 1.38 (1.21, 1.56) | 1.35 (1.22, 1.50) | 1.20 (1.01, 1.43) |  |
| **Grip strength** |  |  |  | 0.019 |
| High | 1 | 1 | 1 |  |
| Low | 1.21 (1.08, 1.35) | 1.12 (1.04, 1.21) | 1.12 (1.02, 1.23) |  |
| **Air pollution** |  |  |  |  |
| **PM_2.5_** |  |  |  | 0.375 |
| Low | 1 | 1 | 1 |  |
| High | 1.04 (0.96, 1.12) | 1.08 (1.03, 1.14) | 1.01 (0.95, 1.08) |  |

Abbreviations: HR, hazard ratios; HDL, high-density lipoprotein; SEP, socioeconomic and psychosocial risk factors; PM_2.5_, fine particulate matter with diameter <2.5 μm.

Models were adjusted for age, sex, ethnicity, region, family history, and mutually adjusted for individual risk factors. **^*^** *P* for interaction was estimated with the use of likelihood ratio test. *P* values were false discovery rate (FDR) corrected.

# Table S6. Hazard ratios of incident cardiovascular diseases associated with individual modifiable risk factors by age groups of <50 years, 50 to <55 years, 55 to <60 years, 60 to <65 years, and ≥65 years

| **Modifiable factors** | **HR (95% CI)** | | | | | ***P* for interaction** |
| --- | --- | --- | --- | --- | --- | --- |
|  | **<50 years** | **50 to <55 years** | **55 to <60 years** | **60 to <65 years** | **≥65 years** |  |
| **Behavioral cluster** |  |  |  |  |  |  |
| **Tobacco use** |  |  |  |  |  | 0.003 |
| Never or former | 1 | 1 | 1 | 1 | 1 |  |
| Current | 1.26 (1.14, 1.41) | 1.18 (1.07, 1.31) | 1.21 (1.12, 1.31) | 1.18 (1.11, 1.25) | 1.11 (1.04, 1.17) |  |
| **Alcohol use** |  |  |  |  |  | 0.464 |
| Never or moderate | 1 | 1 | 1 | 1 | 1 |  |
| Overdrink | 1.12 (0.93, 1.36) | 1.00 (0.83, 1.20) | 1.00 (0.87, 1.15) | 1.06 (0.95, 1.18) | 1.02 (0.90, 1.16) |  |
| **Diet score** |  |  |  |  |  | 0.364 |
| ≥4 | 1 | 1 | 1 | 1 | 1 |  |
| <4 | 1.02 (0.92, 1.14) | 0.95 (0.86, 1.06) | 1.01 (0.93, 1.10) | 1.11 (1.04, 1.19) | 1.07 (1.00, 1.14) |  |
| **Physical activity** |  |  |  |  |  | 0.385 |
| Active | 1 | 1 | 1 | 1 | 1 |  |
| Inactive | 1.08 (0.95, 1.23) | 1.14 (1.01, 1.28) | 1.12 (1.02, 1.23) | 1.04 (0.97, 1.12) | 1.05 (0.97, 1.14) |  |
| **Sleep pattern** |  |  |  |  |  | 0.121 |
| Healthy | 1 | 1 | 1 | 1 | 1 |  |
| Poor | 1.03 (0.93, 1.16) | 1.01 (0.91, 1.12) | 1.10 (1.01, 1.19) | 1.08 (1.02, 1.15) | 1.00 (0.94, 1.06) |  |
| **Metabolic cluster** |  |  |  |  |  |  |
| **Hypertension** |  |  |  |  |  | <0.001 |
| No | 1 | 1 | 1 | 1 | 1 |  |
| Yes | 1.70 (1.52, 1.90) | 1.53 (1.38, 1.69) | 1.46 (1.34, 1.57) | 1.39 (1.31, 1.48) | 1.39 (1.31, 1.48) |  |
| **Diabetes** |  |  |  |  |  | <0.001 |
| No | 1 | 1 | 1 | 1 | 1 |  |
| Yes | 1.89 (1.52, 2.34) | 1.68 (1.40, 2.02) | 1.60 (1.40, 1.83) | 1.65 (1.50, 1.81) | 1.32 (1.21, 1.45) |  |
| **Non-HDL cholesterol** |  |  |  |  |  | <0.001 |
| Low | 1 | 1 | 1 |  |  |  |
| High | 1.45 (1.30, 1.62) | 1.25 (1.13, 1.39) | 1.15 (1.06, 1.24) | 1.00 (0.94, 1.06) | 0.84 (0.79, 0.90) |  |
| **Waist-to-hip ratio** |  |  |  |  |  | <0.001 |
| Low | 1 | 1 | 1 | 1 | 1 |  |
| High | 1.43 (1.27, 1.61) | 1.32 (1.18, 1.48) | 1.24 (1.14, 1.36) | 1.21 (1.13, 1.29) | 1.22 (1.14, 1.30) |  |
| **SEP cluster** |  |  |  |  |  |  |
| **Education level** |  |  |  |  |  | 0.013 |
| High school or further | 1 | 1 | 1 | 1 | 1 |  |
| Less than high school | 1.13 (1.02, 1.26) | 1.14 (1.03, 1.26) | 1.14 (1.05, 1.23) | 1.07 (1.01, 1.14) | 1.04 (0.98, 1.11) |  |
| **Household income (￡)** |  |  |  |  |  | <0.001 |
| High | 1 | 1 | 1 |  |  |  |
| Low | 1.22 (1.08, 1.37) | 1.37 (1.23, 1.53) | 1.10 (1.01, 1.19) | 1.14 (1.07, 1.21) | 1.07 (1.00, 1.15) |  |
| **Depression** |  |  |  |  |  | 0.014 |
| No | 1 | 1 | 1 | 1 | 1 |  |
| Yes | 1.48 (1.23, 1.76) | 1.25 (1.04, 1.51) | 1.59 (1.37, 1.85) | 1.17 (1.01, 1.36) | 1.20 (1.00, 1.43) |  |
| **Grip strength** |  |  |  |  |  | <0.001 |
| High | 1 | 1 | 1 | 1 | 1 |  |
| Low | 1.36 (1.16, 1.60) | 1.12 (0.96, 1.30) | 1.03 (0.91, 1.17) | 1.18 (1.07, 1.29) | 1.14 (1.04, 1.25) |  |
| **Air pollution** |  |  |  |  |  |  |
| **PM_2.5_** |  |  |  |  |  | 0.549 |
| Low | 1 | 1 | 1 | 1 | 1 |  |
| High | 1.03 (0.93, 1.15) | 1.03 (0.93, 1.15) | 1.04 (0.96, 1.13) | 1.09 (1.03, 1.16) | 1.02 (0.95, 1.08) |  |

Abbreviations: HR, hazard ratios; HDL, high-density lipoprotein; SEP, socioeconomic and psychosocial risk factors; PM_2.5_, fine particulate matter with diameter <2.5 μm.

Models were adjusted for age, sex, ethnicity, region, family history, and mutually adjusted for individual risk factors. **^*^** *P* for interaction was estimated with the use of likelihood ratio test. *P* values were false discovery rate (FDR) corrected.

# Table S7. Associations between modifiable risk factors and cardiovascular diseases in overall population stratified by length of follow-up

| **Risk factors** | **Whole sample** | **Follow-up time** | |
| --- | --- | --- | --- |
|  |  | **≥5 years** | **≥10 years** |
| Smoking | 1.16 (1.13, 1.20) | 1.15 (1.11, 1.19) | 1.18 (1.11, 1.25) |
| Alcohol consumption | 1.05 (0.98, 1.12) | 1.08 (1.01, 1.16) | 1.03 (0.92, 1.16) |
| Unhealthy diet | 1.06 (1.03, 1.10) | 1.05 (1.01, 1.10) | 1.02 (0.96, 1.09) |
| Physical inactivity | 1.08 (1.03, 1.12) | 1.08 (1.03, 1.13) | 1.06 (0.98, 1.14) |
| Unhealthy sleep | 1.05 (1.02, 1.09) | 1.04 (1.00, 1.08) | 1.08 (1.01, 1.15) |
| Hypertension | 1.44 (1.40, 1.49) | 1.39 (1.33, 1.44) | 1.36 (1.28, 1.45) |
| Diabetes | 1.52 (1.44, 1.60) | 1.49 (1.39, 1.58) | 1.38 (1.24, 1.54) |
| Non-HDL cholesterol | 1.06 (1.02, 1.09) | 1.00 (0.97, 1.04) | 0.97 (0.91, 1.03) |
| Obesity | 1.25 (1.21, 1.30) | 1.25 (1.20, 1.31) | 1.22 (1.14, 1.30) |
| Less education | 1.09 (1.05, 1.12) | 1.07 (1.03, 1.12) | 1.10 (1.03, 1.17) |
| Low household income | 1.12 (1.09, 1.17) | 1.10 (1.06, 1.15) | 1.11 (1.04, 1.19) |
| Depression | 1.35 (1.26, 1.45) | 1.32 (1.21, 1.44) | 1.22 (1.05, 1.40) |
| Low grip strength | 1.16 (1.10, 1.22) | 1.15 (1.09, 1.22) | 1.13 (1.03, 1.24) |
| High air pollution | 1.05 (1.02, 1.09) | 1.05 (1.00, 1.09) | 1.09 (1.02, 1.16) |

Abbreviations: HDL, high-density lipoprotein

# Table S8. Associations between 14 modifiable factors and incident cardiovascular disease in overall, middle-aged (38 to <50 years), quinquagenarian (50 to <60 years) and the elderly (≥60 years) groups when excluding age from the regression model

| **Exposure** | **Overall** | | **Middle-aged**  **(38 to <50 years)** | | **Quinquagenarian**  **(50 to <60 years)** | | **The elderly**  **(≥60 years)** | |
| --- | --- | --- | --- | --- | --- | --- | --- | --- |
|  | **HR and 95% CI** | ***P* value** | **HR and 95% CI** | ***P* value** | **HR and 95% CI** | ***P* value** | **HR and 95% CI** | ***P* value** |
| Tobacco use | 1.26 (1.22, 1.30) | <0.001 | 1.26 (1.14, 1.41) | <0.001 | 1.22 (1.15, 1.30) | <0.001 | 1.15 (1.10, 1.20) | <0.001 |
| Alcohol use | 1.01 (0.95, 1.07) | 0.800 | 1.12 (0.93, 1.36) | 0.240 | 1.00 (0.89, 1.12) | 0.937 | 1.03 (0.95, 1.12) | 0.468 |
| Unhealthy diet | 0.94 (0.91, 0.97) | <0.001 | 1.02 (0.92, 1.14) | 0.656 | 0.97 (0.91, 1.03) | 0.340 | 1.08 (1.03, 1.13) | 0.001 |
| Physical inactivity | 1.05 (1.00, 1.09) | 0.031 | 1.08 (0.95, 1.23) | 0.233 | 1.13 (1.05, 1.22) | 0.001 | 1.04 (0.98, 1.09) | 0.205 |
| Unhealthy sleep pattern | 1.02 (0.99, 1.06) | 0.175 | 1.03 (0.93, 1.16) | 0.549 | 1.06 (1.00, 1.13) | 0.069 | 1.03 (0.99, 1.07) | 0.169 |
| Hypertension | 1.82 (1.76, 1.89) | <0.001 | 1.70 (1.52, 1.90) | <0.001 | 1.52 (1.43, 1.62) | <0.001 | 1.43 (1.37, 1.50) | <0.001 |
| Diabetes | 1.60 (1.51, 1.69) | <0.001 | 1.89 (1.52, 2.34) | <0.001 | 1.64 (1.47, 1.83) | <0.001 | 1.48 (1.39, 1.58) | <0.001 |
| High non-HDL-C | 1.05 (1.02, 1.09) | 0.004 | 1.45 (1.30, 1.62) | <0.001 | 1.19 (1.12, 1.27) | <0.001 | 0.91 (0.87, 0.95) | <0.001 |
| High waist-to-hip ratio | 1.36 (1.31, 1.41) | <0.001 | 1.43 (1.27, 1.61) | <0.001 | 1.28 (1.19, 1.37) | <0.001 | 1.22 (1.17, 1.28) | <0.001 |
| Less education level | 1.07 (1.04, 1.11) | <0.001 | 1.13 (1.02, 1.26) | 0.024 | 1.13 (1.06, 1.20) | <0.001 | 1.06 (1.02, 1.11) | 0.004 |
| Low income | 1.56 (1.51, 1.61) | <0.001 | 1.22 (1.08, 1.37) | 0.001 | 1.23 (1.15, 1.31) | <0.001 | 1.18 (1.13, 1.23) | <0.001 |
| Depression | 1.08 (1.01, 1.17) | 0.029 | 1.48 (1.23, 1.76) | <0.001 | 1.40 (1.25, 1.58) | <0.001 | 1.14 (1.02, 1.28) | 0.022 |
| Low grip strength | 1.44 (1.37, 1.51) | <0.001 | 1.36 (1.16, 1.60) | <0.001 | 1.10 (1.00, 1.21) | 0.050 | 1.20 (1.13, 1.28) | <0.001 |
| High PM_2.5_ concentration | 0.97 (0.94, 1.00) | 0.076 | 1.03 (0.93, 1.15) | 0.577 | 1.03 (0.97, 1.10) | 0.367 | 1.04 (1.00, 1.09) | 0.049 |

Abbreviations: CI, confidence interval; HDL, high-density lipoprotein; PM_2.5_, fine particulate matter with diameter <2.5 μm.

# Table S9. Hazard ratios and 95% CI of incident cardiovascular diseases associated with individual modifiable risk factors by age groups using multiple imputation to impute data

| **Modifiable factors** | **HR (95% CI)** | | | ***P* for interaction^*^** |
| --- | --- | --- | --- | --- |
|  | **Middle-aged**  **(40 to <50 years)** | **Quinquagenarian**  **(50 to <60 years)** | **The elderly**  **(≥60 years)** |  |
| **Behavioral cluster** |  |  |  |  |
| **Tobacco use** |  |  |  | <0.001 |
| Never or former | 1 | 1 | 1 |  |
| Current | 1.27 (1.19, 1.37) | 1.26 (1.21, 1.32) | 1.16 (1.13, 1.19) |  |
| **Alcohol use** |  |  |  | 0.112 |
| Never or moderate | 1 | 1 | 1 |  |
| Overdrink | 1.10 (0.98, 1.25) | 0.97 (0.90, 1.05) | 1.04 (0.99, 1.10) |  |
| **Diet score** |  |  |  | 0.106 |
| ≥4 | 1 | 1 | 1 |  |
| <4 | 1.05 (0.97, 1.12) | 1.04 (0.99, 1.08) | 1.07 (1.04, 1.10) |  |
| **Physical activity** |  |  |  | 0.025 |
| Active | 1 | 1 | 1 |  |
| Inactive | 1.08 (0.99, 1.17) | 1.14 (1.09, 1.20) | 1.06 (1.03, 1.10) |  |
| **Sleep pattern** |  |  |  | 0.002 |
| Healthy | 1 | 1 | 1 |  |
| Poor | 1.08 (1.00, 1.16) | 1.09 (1.04, 1.14) | 1.01 (0.98, 1.04) |  |
| **Metabolic cluster** |  |  |  |  |
| **Hypertension** |  |  |  | <0.001 |
| No | 1 | 1 | 1 |  |
| Yes | 1.65 (1.53, 1.78) | 1.49 (1.43, 1.55) | 1.37 (1.33, 1.41) |  |
| **Diabetes** |  |  |  | <0.001 |
| No | 1 | 1 | 1 |  |
| Yes | 1.85 (1.61, 2.12) | 1.56 (1.45, 1.68) | 1.41 (1.35, 1.47) |  |
| **Non-HDL cholesterol** |  |  |  | <0.001 |
| Low | 1 | 1 | 1 |  |
| High | 1.23 (1.14, 1.33) | 1.07 (1.02, 1.12) | 0.95 (0.92, 0.98) |  |
| **Waist-to-hip ratio** |  |  |  | <0.001 |
| Low | 1 | 1 | 1 |  |
| High | 1.44 (1.33, 1.56) | 1.27 (1.21, 1.34) | 1.23 (1.19, 1.27) |  |
| **SEP cluster** |  |  |  |  |
| **Education level** |  |  |  | 0.001 |
| High school or further | 1 | 1 | 1 |  |
| Less than high school | 1.13 (1.05, 1.21) | 1.11 (1.07, 1.16) | 1.06 (1.03, 1.09) |  |
| **Household income (￡)** |  |  |  | 0.002 |
| High | 1 | 1 | 1 |  |
| Low | 1.18 (1.09, 1.28) | 1.21 (1.16, 1.27) | 1.10 (1.07, 1.13) |  |
| **Depression** |  |  |  | 0.001 |
| No | 1 | 1 | 1 |  |
| Yes | 1.37 (1.23, 1.54) | 1.43 (1.33, 1.54) | 1.22 (1.15, 1.31) |  |
| **Grip strength** |  |  |  | <0.001 |
| High | 1 | 1 | 1 |  |
| Low | 1.30 (1.17, 1.44) | 1.05 (0.99, 1.12) | 1.16 (1.11, 1.20) |  |
| **Air pollution** |  |  |  | 0.732 |
| **PM_2.5_** |  |  |  |  |
| Low | 1 | 1 | 1 |  |
| High | 1.01 (0.94, 1.09) | 1.05 (1.00, 1.09) | 1.06 (1.03, 1.09) |  |

Abbreviations: HR, hazard ratios; CI, confidence interval; HDL, high-density lipoprotein; SEP, socioeconomic and psychosocial risk factors; PM_2.5_, fine particulate matter with diameter <2.5 μm.

*^*^P* for interaction was estimated with the use of likelihood ratio test. *P* values were false discovery rate (FDR) corrected.

# Table S10. Hazard ratios and 95% CI of incident cardiovascular diseases associated with individual modifiable risk factors by age groups using Fine & Gray’s competing risk model

| **Modifiable factors** | **HR (95% CI)** | | | ***P* for interaction^*^** |
| --- | --- | --- | --- | --- |
|  | **Middle-aged**  **(40 to <50 years)** | **Quinquagenarian**  **(50 to <60 years)** | **The elderly**  **(≥60 years)** |  |
| **Behavioral cluster** |  |  |  |  |
| **Tobacco use** |  |  |  | 0.126 |
| Never or former | 1 | 1 | 1 |  |
| Current | 1.35 (1.12, 1.62) | 1.32 (1.19, 1.47) | 1.28 (1.19, 1.37) |  |
| **Alcohol use** |  |  |  | 0.412 |
| Never or moderate | 1 | 1 | 1 |  |
| Overdrink | 1.35 (0.98, 1.86) | 1.23 (1.02, 1.49) | 1.13 (0.99, 1.30) |  |
| **Diet score** |  |  |  | 0.324 |
| ≥4 | 1 | 1 | 1 |  |
| <4 | 0.99 (0.82, 1.20) | 1.23 (1.09, 1.37) | 1.20 (1.11, 1.29) |  |
| **Physical activity** |  |  |  | 0.185 |
| Active | 1 | 1 | 1 |  |
| Inactive | 1.19 (0.95, 1.47) | 1.08 (0.95, 1.23) | 1.20 (1.10, 1.31) |  |
| **Sleep pattern** |  |  |  | 0.008 |
| Healthy | 1 | 1 | 1 |  |
| Poor | 1.28 (1.05, 1.56) | 1.11 (0.99, 1.25) | 0.96 (0.89, 1.03) |  |
| **Metabolic cluster** |  |  |  |  |
| **Hypertension** |  |  |  | 0.001 |
| No | 1 | 1 | 1 |  |
| Yes | 1.21 (0.98, 1.49) | 1.21 (1.09, 1.35) | 0.97 (0.90, 1.04) |  |
| **Diabetes** |  |  |  | 0.012 |
| No | 1 | 1 | 1 |  |
| Yes | 2.24 (1.57, 3.19) | 1.69 (1.40, 2.04) | 1.39 (1.24, 1.56) |  |
| **Non-HDL cholesterol** |  |  |  | 0.542 |
| Low | 1 | 1 | 1 |  |
| High | 1.00 (0.81, 1.22) | 0.93 (0.83, 1.04) | 0.96 (0.89, 1.03) |  |
| **Waist-to-hip ratio** |  |  |  | 0.037 |
| Low | 1 | 1 | 1 |  |
| High | 1.35 (1.10, 1.66) | 1.15 (1.01, 1.30) | 1.07 (0.99, 1.15) |  |
| **SEP cluster** |  |  |  |  |
| **Education level** |  |  |  | 0.048 |
| High school or further | 1 | 1 | 1 |  |
| Less than high school | 1.21 (1.00, 1.46) | 0.94 (0.84, 1.05) | 1.04 (0.97, 1.12) |  |
| **Household income (￡)** |  |  |  | 0.001 |
| High | 1 | 1 | 1 |  |
| Low | 1.26 (1.03, 1.55) | 1.54 (1.38, 1.73) | 1.21 (1.12, 1.31) |  |
| **Depression** |  |  |  | 0.274 |
| No | 1 | 1 | 1 |  |
| Yes | 1.08 (0.77, 1.53) | 1.28 (1.04, 1.58) | 1.34 (1.12, 1.60) |  |
| **Grip strength** |  |  |  | 0.037 |
| High | 1 | 1 | 1 |  |
| Low | 1.24 (0.97, 1.59) | 1.44 (1.21, 1.71) | 1.16 (1.04, 1.29) |  |
| **Air pollution** |  |  |  | 0.235 |
| **PM_2.5_** |  |  |  |  |
| Low | 1 | 1 | 1 |  |
| High | 1.09 (0.91, 1.32) | 1.17 (1.04, 1.30) | 1.11 (1.03, 1.20) |  |

Abbreviations: HR, hazard ratios; CI, confidence interval; HDL, high-density lipoprotein; SEP, socioeconomic and psychosocial risk factors; PM_2.5_, fine particulate matter with diameter <2.5 μm.

*^*^P* for interaction was estimated with the use of likelihood ratio test. *P* values were false discovery rate (FDR) corrected.

# Table S11. Additive interaction between age groups and each modifiable risk factors on risk of CVD

|  | Quinquagenarian | | The elderly | |
| --- | --- | --- | --- | --- |
|  | RERIs (95% CIs) | APs (95% CIs) | RERIs (95% CIs) | APs (95% CIs) |
| Smoking | -0.08 (-0.40, 0.24) | -0.06 (-0.31, 0.18) | -0.19 (-0.55, 0.15) | -0.17 (-0.49, 0.15) |
| Alcohol consumption | -0.17 (-0.52, 0.17) | -0.16 (-0.49, 0.17) | -0.17 (-0.55, 0.20) | -0.18 (-0.57, 0.21) |
| Unhealthy diet | -0.08 (-0.37, 0.20) | -0.07 (-0.34, 0.19) | -0.07 (-0.39, 0.25) | -0.06 (-0.38, 0.25) |
| Physical inactivity | 0.03 (-0.26, 0.33) | 0.03 (-0.21, 0.27) | -0.07 (-0.39, 0.25) | -0.07 (-0.39, 0.25) |
| Unhealthy sleep | -0.01 (-0.32, 0.31) | -0.01 (-0.27, 0.27) | -0.07 (-0.41, 0.27) | -0.06 (-0.40, 0.27) |
| Hypertension | -0.19 (-0.58, 0.20) | -0.11 (-0.34, 0.12) | -0.43 (-0.86, -0.01) | -0.30 (-0.61, -0.01) |
| Diabetes | -0.20 (-0.74, 0.34) | -0.11 (-0.43, 0.20) | -0.54 (-1.10, 0.01) | -0.38 (-0.81, 0.03) |
| Non-HDL cholesterol | -0.24 (-0.59, 0.10) | -0.17 (-0.43, 0.08) | -0.61 (-0.99, -0.22) | -0.57 (-0.97, -0.17) |
| Obesity | -0.19 (-0.57, 0.19) | -0.13 (-0.39, 0.14) | -0.47 (-0.91, -0.03) | -0.35 (-0.70, -0.01) |
| Less education | -0.02 (-0.33, 0.28) | -0.02 (-0.26, 0.23) | -0.14 (-0.48, 0.19) | -0.14 (-0.47, 0.18) |
| Low household income | -0.05 (-0.36, 0.26) | -0.04 (-0.29, 0.20) | -0.19 (-0.53, 0.14) | -0.18 (-0.51, 0.14) |
| Depression | -0.03 (-0.45, 0.40) | -0.02 (-0.29, 0.25) | -0.40 (-0.83, 0.04) | -0.34 (-0.75, 0.06) |
| Low grip strength | -0.06 (-0.38, 0.26) | -0.05 (-0.32, 0.21) | 0.14 (-0.17, 0.45) | 0.13 (-0.15, 0.41) |
| High air pollution | 0.01 (-0.27, 0.28) | 0.01 (-0.24, 0.25) | -0.02 (-0.32, 0.29) | -0.02 (-0.32, 0.29) |

Abbreviations: HDL, high-density lipoprotein; RERI, relative excess risk due to interaction; AP, attributable proportion due to interaction.

# Table S12. Hazard ratios (95% CIs) of incident cardiovascular diseases associated with individual modifiable risk factors among female and male

| **Characteristics** | **Female (N=119,252)** | **Male (N=107,507)** | ***P*-value ^*^** |
| --- | --- | --- | --- |
| **Age, years** | 55.29 (7.97) | 55.78 (8.16) | <0.001 |
| **Ethnicity, n (%)** |  |  | 0.888 |
| White | 113672 (95.32) | 102491 (95.33) |  |
| Nonwhite | 5580 (4.68) | 5016 (4.67) |  |
| **Behavioral cluster** |  |  |  |
| **Tobacco use, n (%)** |  |  | <0.001 |
| Never or former | 71665 (60.10) | 54149 (50.37) |  |
| Current | 47587 (39.90) | 53358 (49.63) |  |
| **Alcohol use, n (%)** |  |  | <0.001 |
| Never or moderate | 113457 (95.14) | 97961 (91.12) |  |
| Overdrink | 5795 (4.86) | 9546 (8.88) |  |
| **Diet score** |  |  | <0.001 |
| <4 | 72986 (77.85) | 51989 (60.78) |  |
| ≥4 | 20762 (22.15) | 33549 (39.22) |  |
| **Physical activity** |  |  | 0.231 |
| Active | 81572 (81.78) | 78051 (81.56) |  |
| Inactive | 18179 (18.22) | 17641 (18.44) |  |
| **Sleep pattern** |  |  | 0.018 |
| Healthy | 42446 (35.59) | 37754 (35.12) |  |
| Poor | 76806 (64.41) | 69753 (64.88) |  |
| **Metabolic cluster** |  |  |  |
| **Hypertension** |  |  | <0.001 |
| No | 77458 (64.95) | 57248 (53.25) |  |
| Yes | 41794 (35.05) | 50259 (46.75) |  |
| **Diabetes** |  |  | <0.001 |
| No | 109072 (96.41) | 95655 (93.50) |  |
| Yes | 4067 (3.59) | 6651 (6.50) |  |
| **Non-HDL cholesterol** |  |  | <0.001 |
| Low | 80786 (67.74) | 70408 (65.49) |  |
| High | 38466 (32.26) | 37099 (34.51) |  |
| **Waist-to-hip ratio** |  |  | <0.001 |
| Low | 84512 (70.87) | 33049 (30.74) |  |
| High | 34740 (29.13) | 74458 (69.26) |  |
| **SEP cluster** |  |  |  |
| **Education level** |  |  | 0.001 |
| High school or further | 60600 (50.96) | 55371 (51.66) |  |
| Less than high school | 58327 (49.04) | 51811 (48.34) |  |
| **Household income** |  |  | <0.001 |
| High | 63371 (53.24) | 64590 (60.20) |  |
| Low | 55647 (46.76) | 42697 (39.80) |  |
| **Depression** |  |  | <0.001 |
| No | 112531 (94.36) | 102469 (95.31) |  |
| Yes | 6721 (5.64) | 5038 (4.69) |  |
| **Grip strength** |  |  | <0.001 |
| High | 33182 (27.83) | 100433 (93.42) |  |
| Low | 86070 (72.17) | 7074 (6.58) |  |
| **Air pollution** |  |  |  |
| **PM_2.5_ (μg/m^3^)** | 9.96 (1.04) | 9.96 (1.06) | 0.462 |

Abbreviations: HDL, high-density lipoprotein; SEP, socioeconomic and psychosocial risk factors; PM_2.5_, fine particulate matter with diameter <2.5 μm.

**^*^***P* values are based on T test for continuous variables or χ² test for categorical variables.


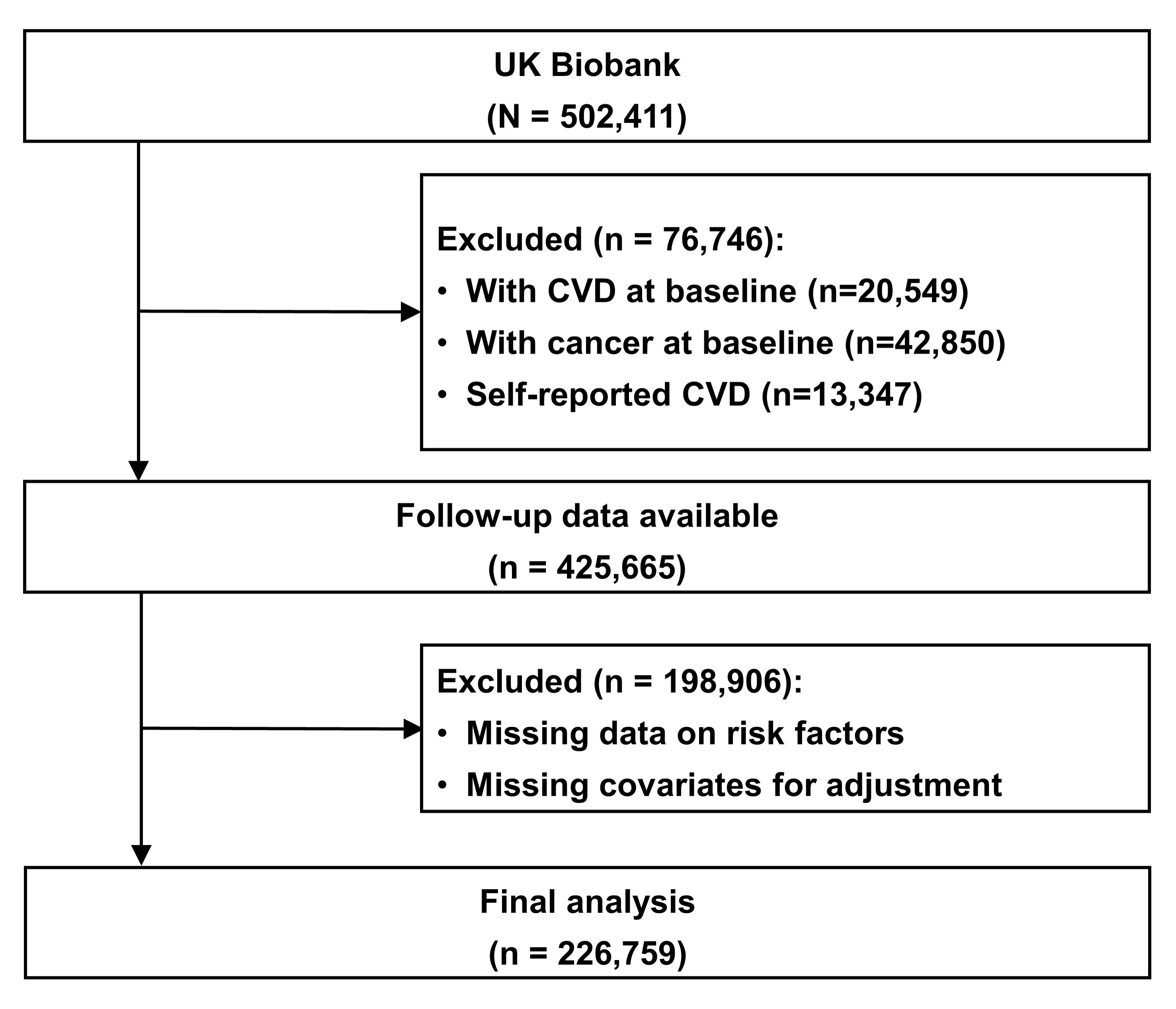


# Figure S1. Flowchart of the study participants included and excluded in study

CVD: cardiovascular disease.

**
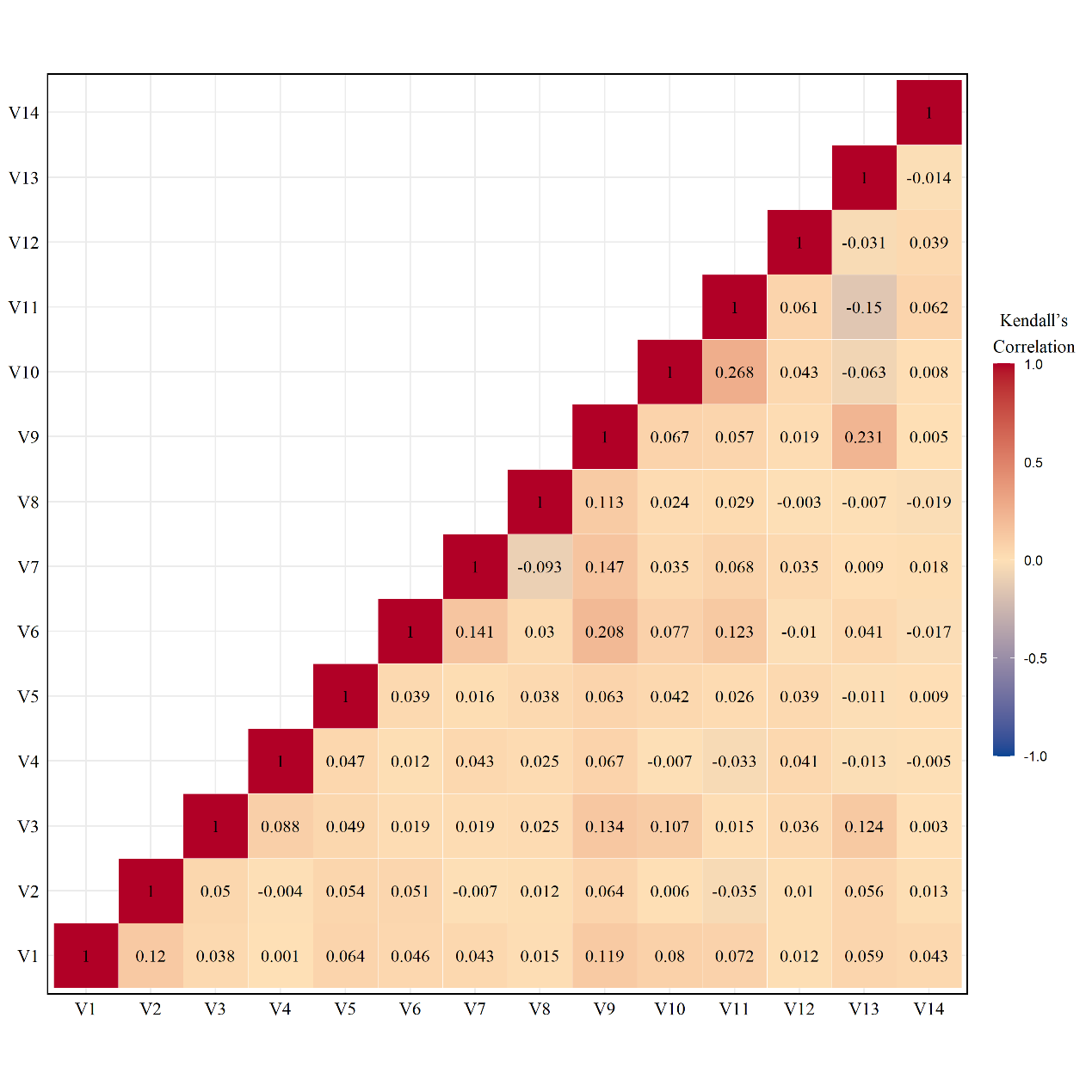
** Figure S2. The correlation matrix between each modifiable risk factor using Kendall’s correlation. V1, smoking; V2, alcohol consumption; V3, diet; V4, physical activity; V5, sleep; V6, hypertension; V7, diabetes; V8, non-HDL cholesterol; V9, waist-to-hip ratio; V10, education; V11, household income; V12, depression; V13, grip strength; V14, air pollution.


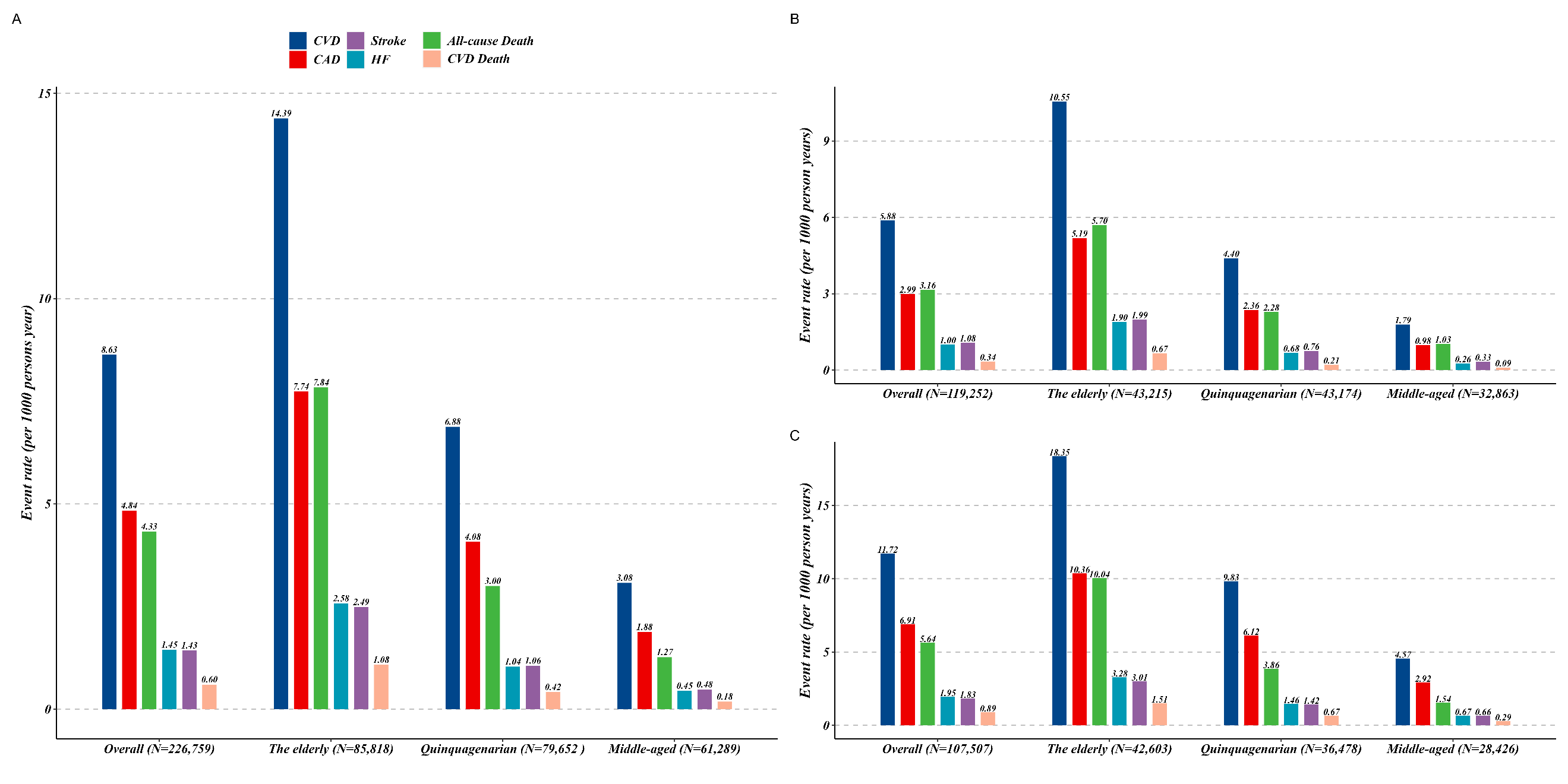
Figure S3. The event rates of cardiovascular disease events and mortality stratified by age groups in overall population (A), female (B) and male (C)**.** Abbreviations: CVD, cardiovascular disease; CAD, coronary artery disease; HF, heart failure.


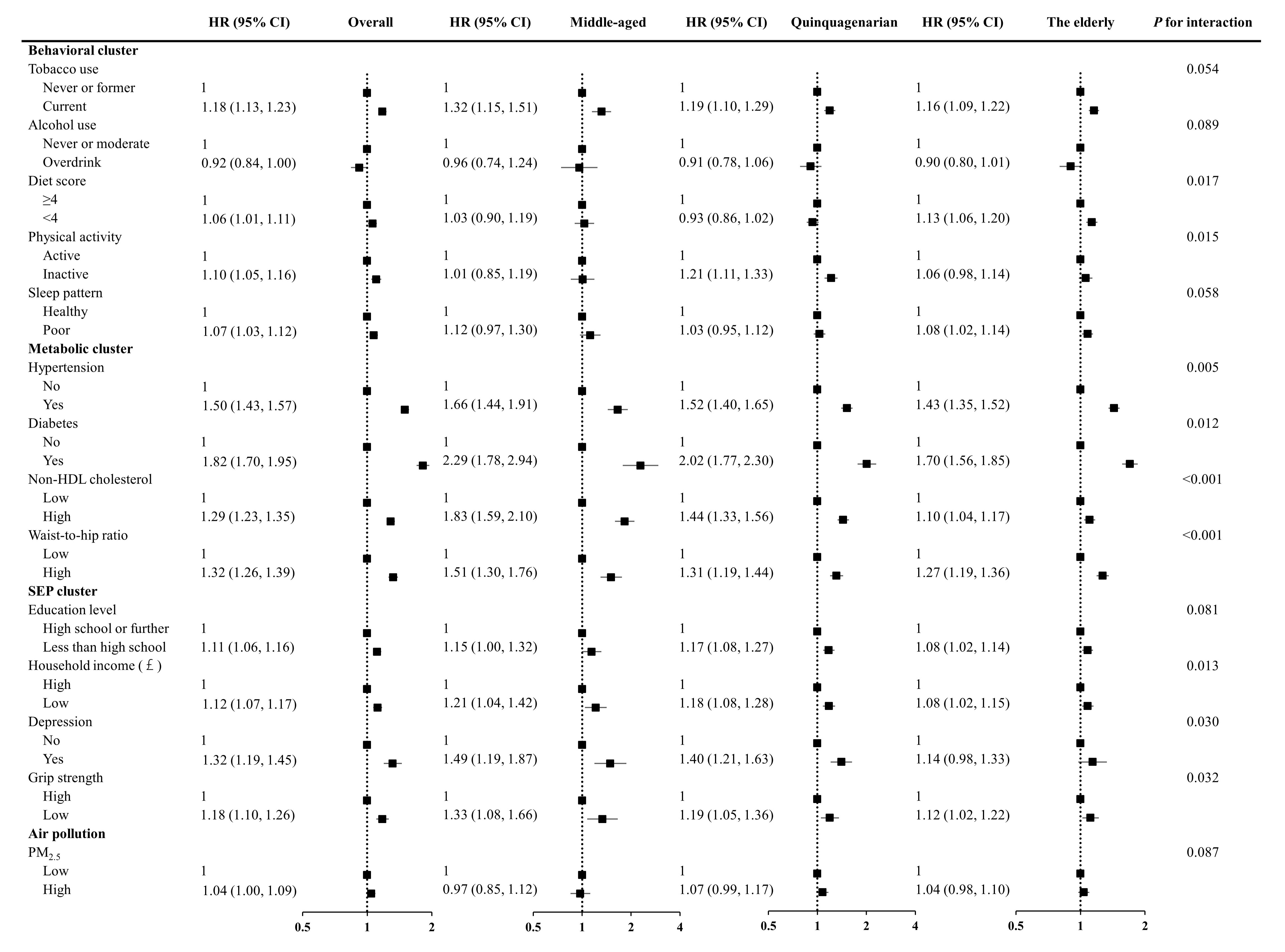
Figure S4. Associations between 14 modifiable factors and incident coronary artery disease in middle-aged (38 to <50 years), quinquagenarian (50 to <60 years) and the elderly (≥60 years) groups.

Models were adjusted for age, sex, ethnicity, region, family history, and mutually adjusted for individual risk factors. *P* for interaction was estimated with the use of likelihood ratio test. *P* values were false discovery rate (FDR) corrected.

Abbreviations: HR, hazard ratios; HDL, high-density lipoprotein; SEP, socioeconomic and psychosocial risk factors; PM_2.5_, fine particulate matter with diameter <2.5 μm.


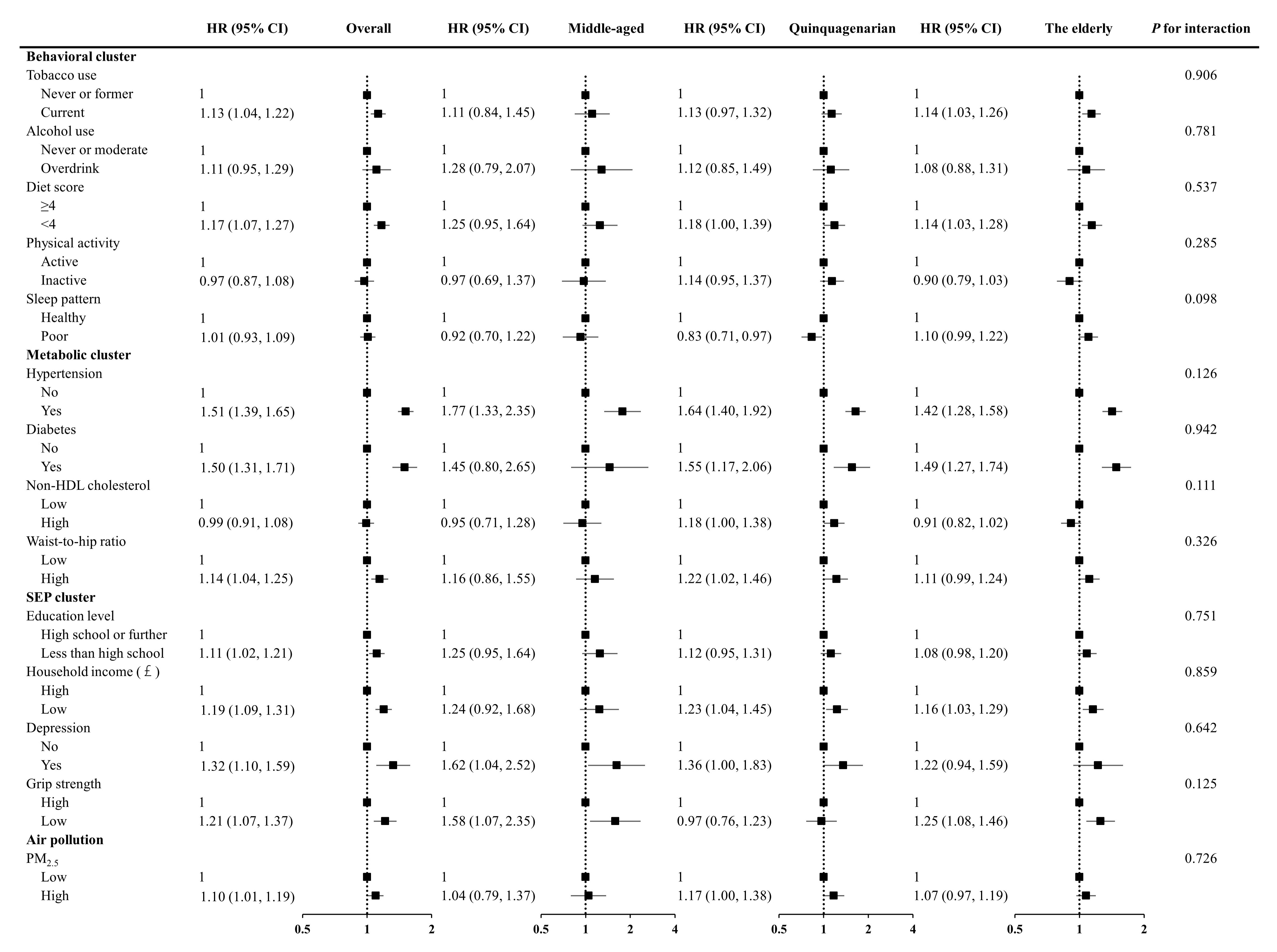
Figure S5. Associations between 14 modifiable factors and incident stroke in middle-aged (38 to <50 years), quinquagenarian (50 to <60 years) and the elderly (≥60 years) groups.

Models were adjusted for age, sex, ethnicity, region, family history and mutually adjusted for individual risk factors. *P* for interaction was estimated with the use of likelihood ratio test. *P* values were false discovery rate (FDR) corrected.

Abbreviations: HR, hazard ratios; HDL, high-density lipoprotein; SEP, socioeconomic and psychosocial risk factors; PM_2.5_, fine particulate matter with diameter <2.5 μm.


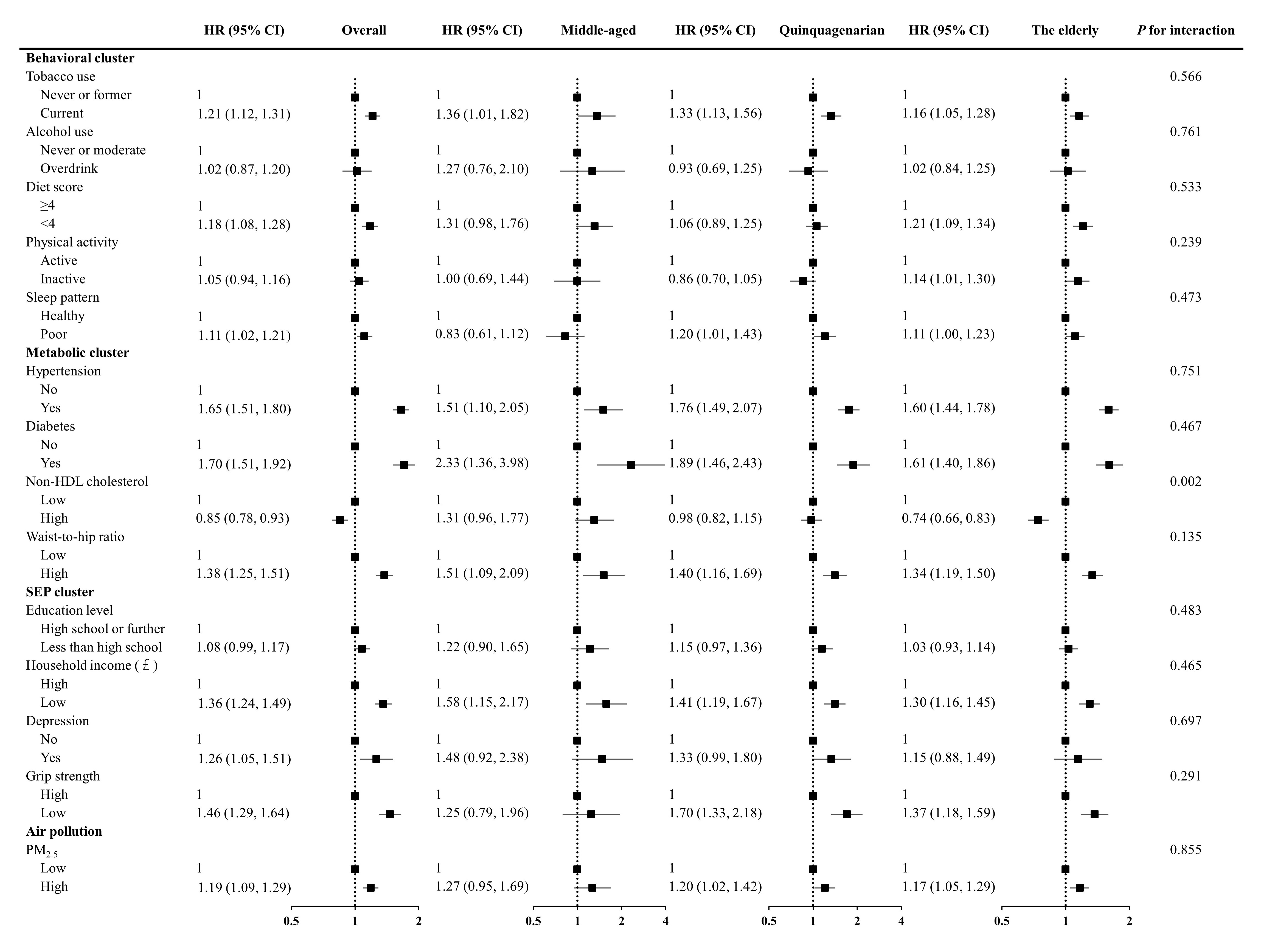
Figure S6. Associations between 14 modifiable factors and incident heart failure in middle-aged (38 to <50 years), quinquagenarian (50 to <60 years) and the elderly (≥60 years) groups**.**

Models were adjusted for age, sex, ethnicity, region, family history, and mutually adjusted for individual risk factors. *P* for interaction was estimated with the use of likelihood ratio test. *P* values were false discovery rate (FDR) corrected.

Abbreviations: HR, hazard ratios; HDL, high-density lipoprotein; SEP, socioeconomic and psychosocial risk factors; PM_2.5_, fine particulate matter with diameter <2.5 μm.


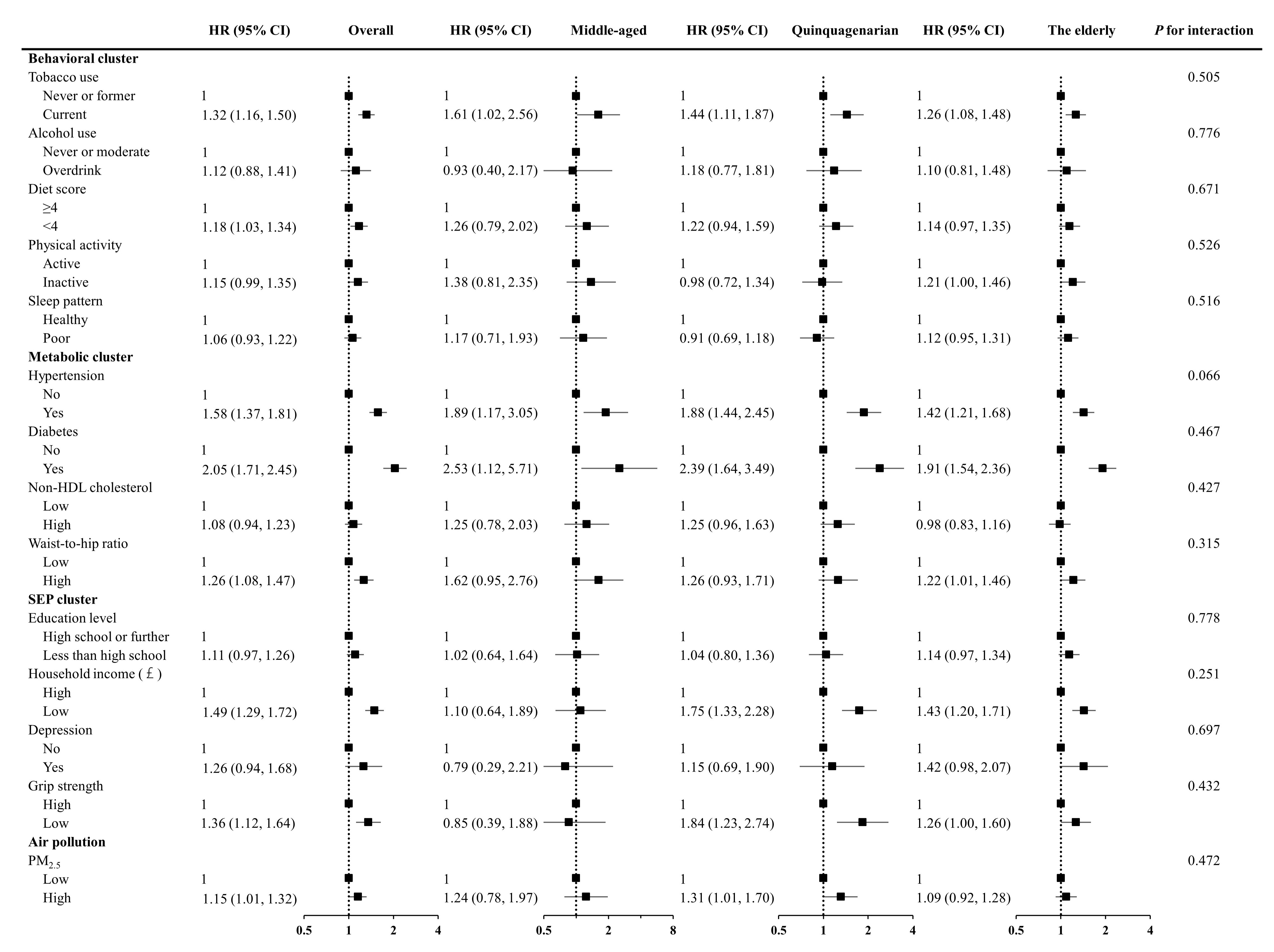
Figure S7. Associations between 14 modifiable factors and cardiovascular mortality in middle-aged (38 to <50 years), quinquagenarian (50 to <60 years) and the elderly (≥60 years) groups**.**

Models were adjusted for age, sex, ethnicity, region, family history and mutually adjusted for individual risk factors. *P* for interaction was estimated with the use of likelihood ratio test. *P* values were false discovery rate (FDR) corrected.

Abbreviations: HR, hazard ratios; HDL, high-density lipoprotein; SEP, socioeconomic and psychosocial risk factors; PM_2.5_, fine particulate matter with diameter <2.5 μm.


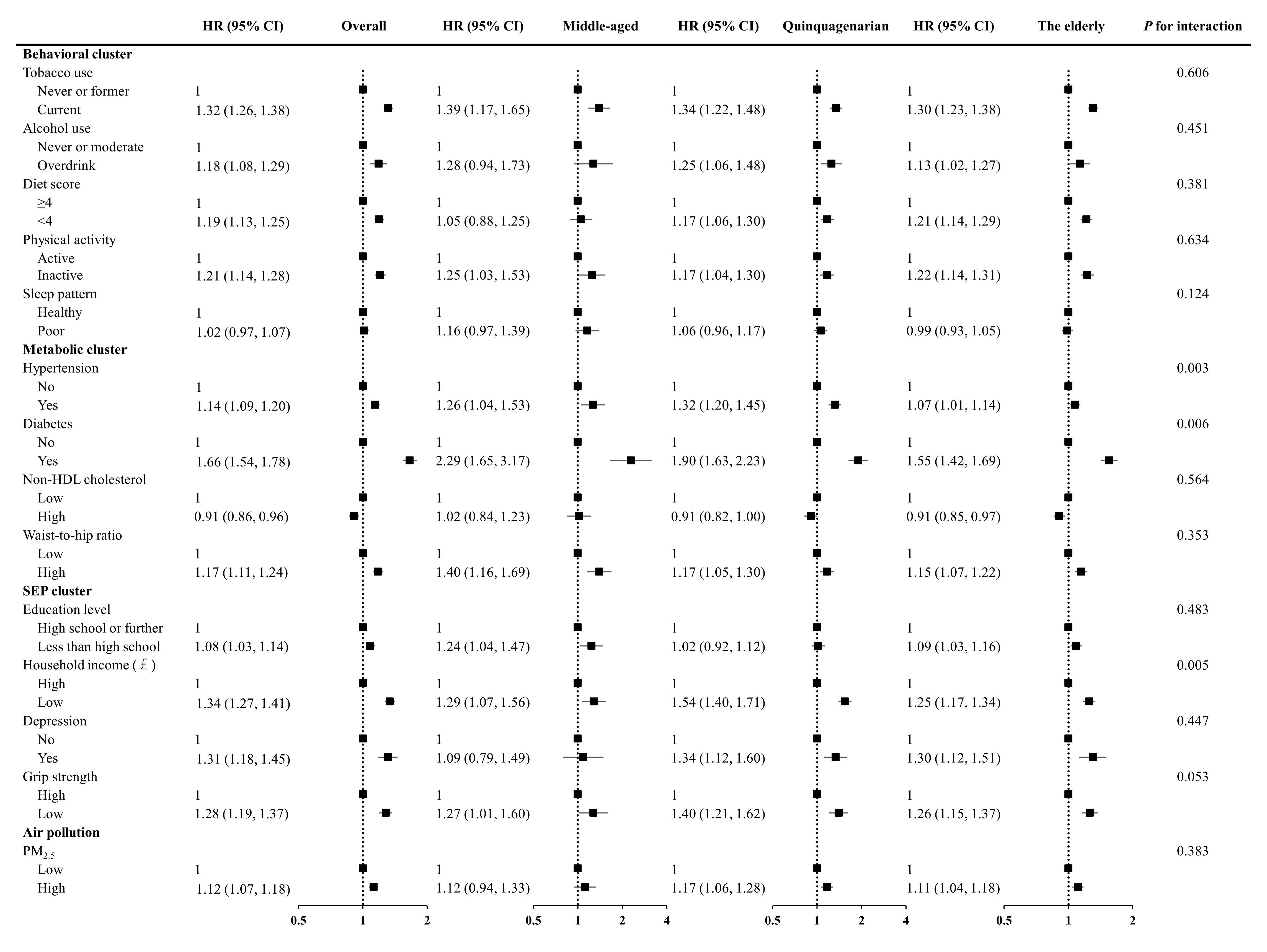
Figure S8. Associations between 14 modifiable factors and all-cause mortality in middle-aged (38 to <50 years), quinquagenarian (50 to <60 years) and the elderly (≥60 years) groups**.**

Models were adjusted for age, sex, ethnicity, region, family history, and mutually adjusted for individual risk factors. *P* for interaction was estimated with the use of likelihood ratio test. *P* values were false discovery rate (FDR) corrected.

Abbreviations: HR, hazard ratios; HDL, high-density lipoprotein; SEP, socioeconomic and psychosocial risk factors; PM_2.5_, fine particulate matter with diameter <2.5 μm.


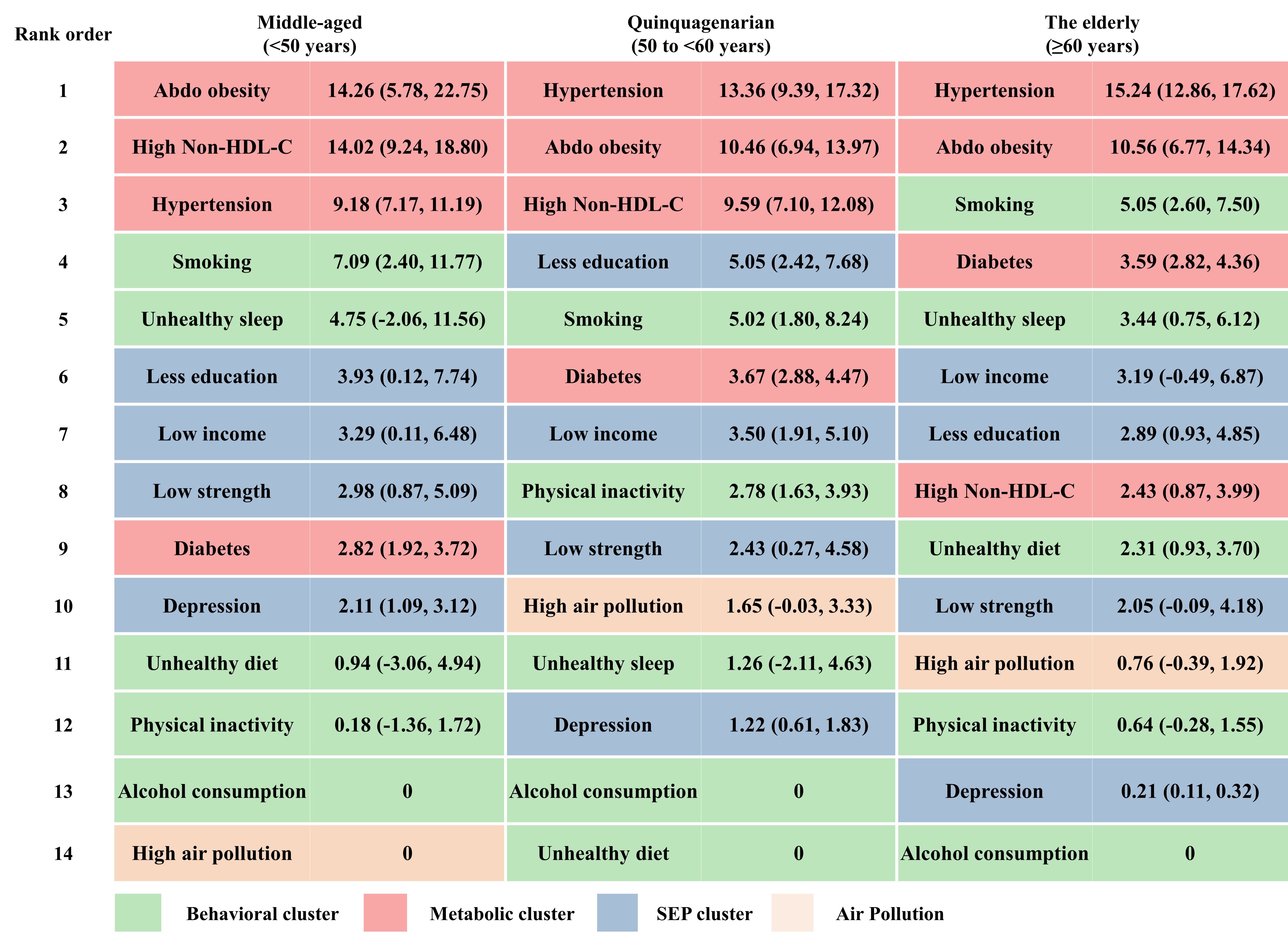
Figure S9. Ranking the population attributable fractions for incident coronary artery disease associated with the 14 modifiable risk factors among age groups**.**

Models were adjusted for age, sex, ethnicity, region, family history, and mutually adjusted for individual risk factors. Estimated PAFs for modifiable risk factors were truncated at a lower limit of 0, as this is the lowest threshold to show a relationship with increased risk. Abbreviations: Abdo obesity, abdominal obesity; Non-HDL-C, non-high-density lipoprotein cholesterol; SEP, socioeconomic and psychosocial risk factors; PAF, population attributable fractions.


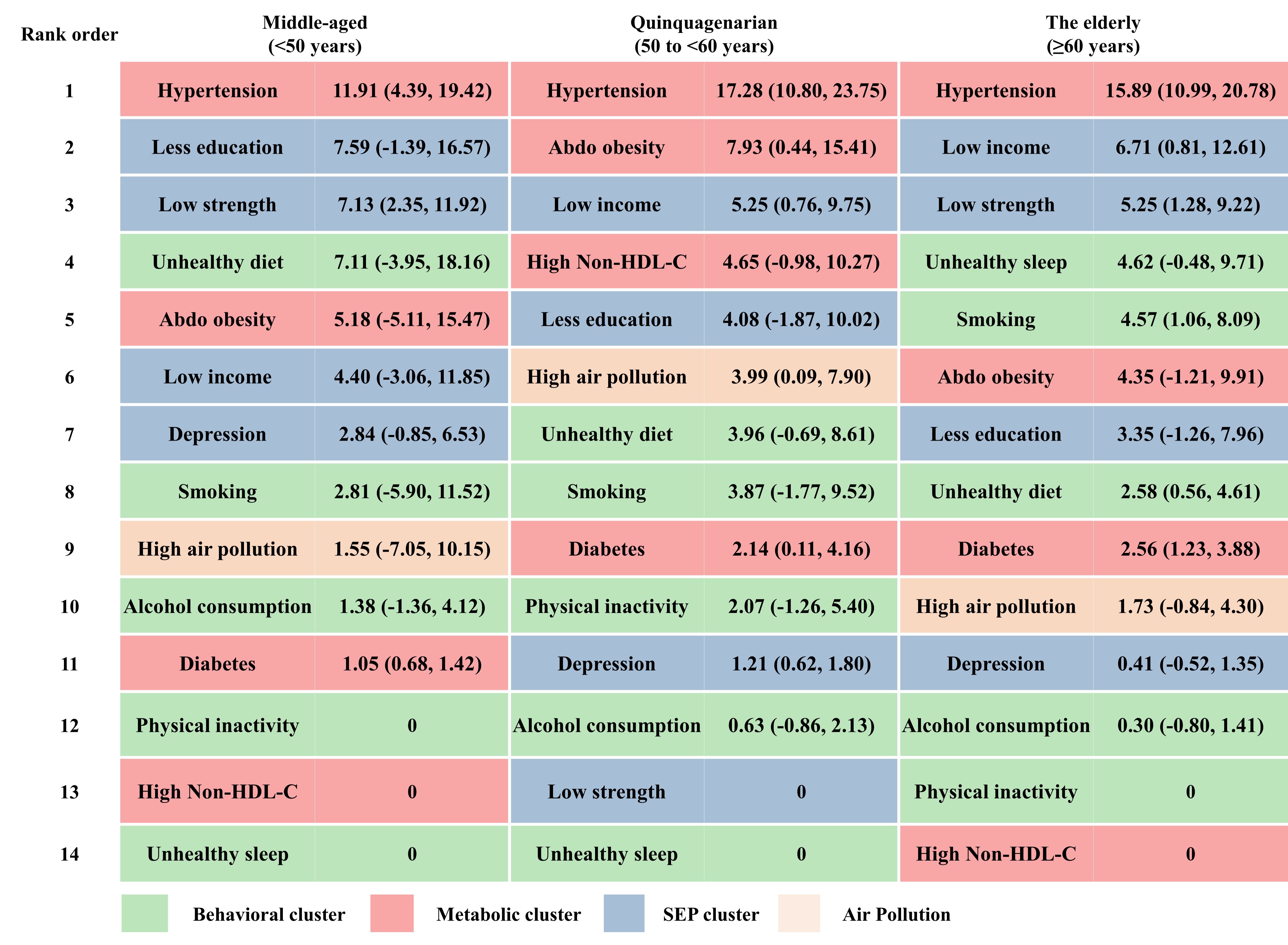
Figure S10. Ranking the population attributable fractions for incident stroke associated with the 14 modifiable risk factors among age groups**.**

Models were adjusted for age, sex, ethnicity, region, family history, and mutually adjusted for individual risk factors. Estimated PAFs for modifiable risk factors were truncated at a lower limit of 0, as this is the lowest threshold to show a relationship with increased risk. Abbreviations: Abdo obesity, abdominal obesity; Non-HDL-C, non-high-density lipoprotein cholesterol; SEP, socioeconomic and psychosocial risk factors; PAF, population attributable fractions.


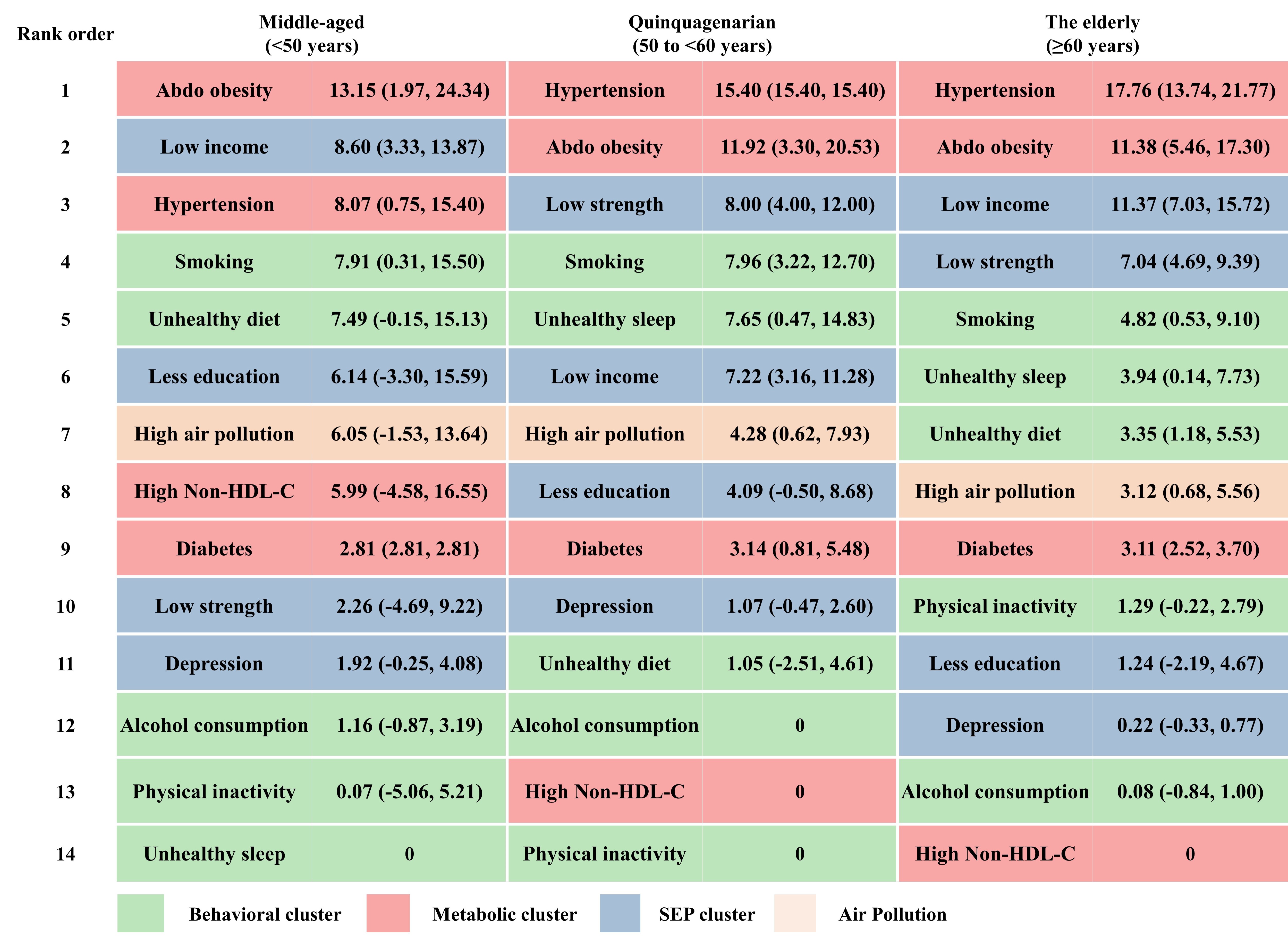
Figure S11. Ranking the population attributable fractions for incident heart failure associated with the 14 modifiable risk factors among age groups.

Models were adjusted for age, sex, ethnicity, region, family history, and mutually adjusted for individual risk factors. Estimated PAFs for modifiable risk factors were truncated at a lower limit of 0, as this is the lowest threshold to show a relationship with increased risk. Abbreviations: Abdo obesity, abdominal obesity; Non-HDL-C, non-high-density lipoprotein cholesterol; SEP, socioeconomic and psychosocial risk factors; PAF, population attributable fractions.


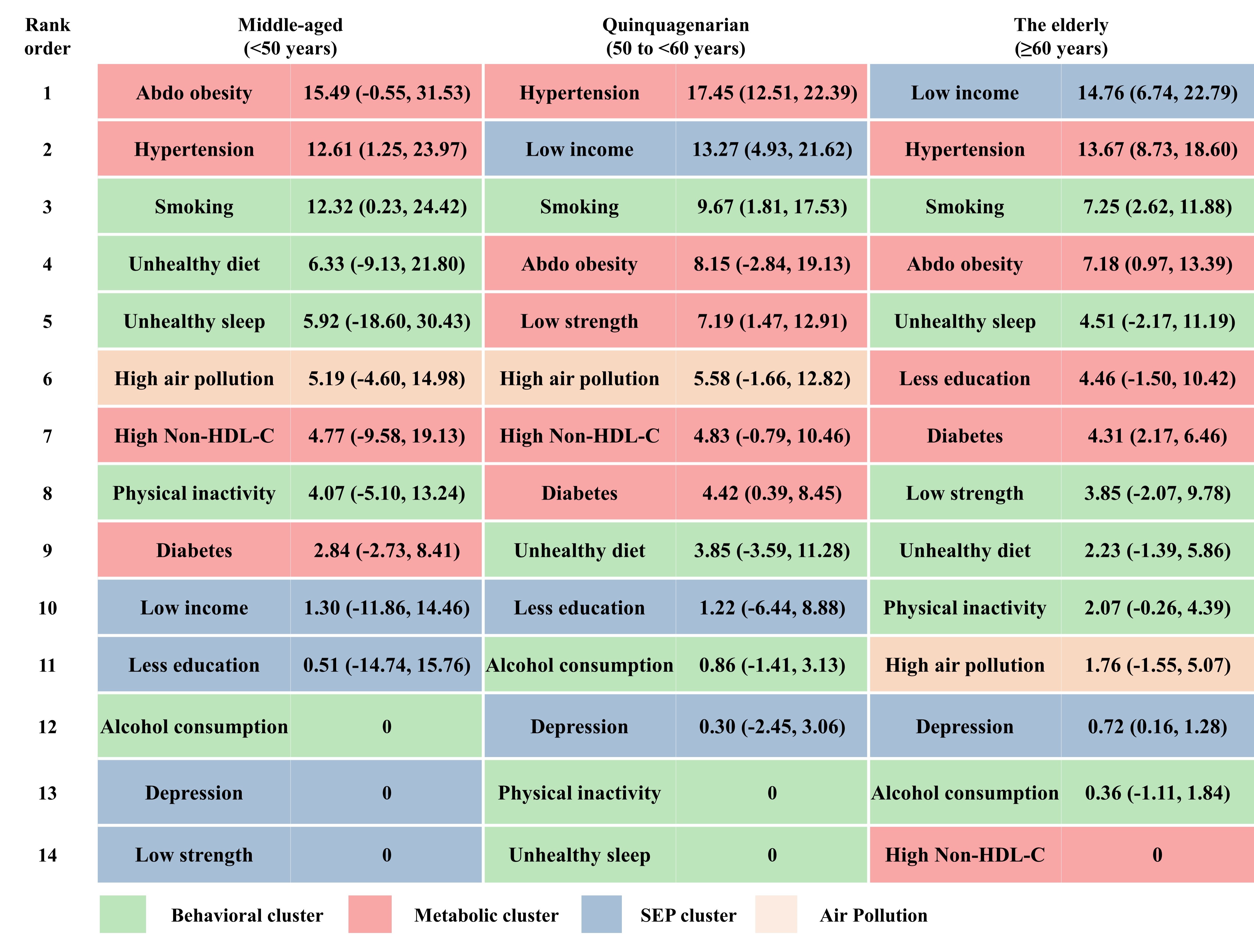
Figure S12. Ranking the population attributable fractions for cardiovascular mortality associated with the 14 modifiable risk factors among age groups**.**

Models were adjusted for age, sex, ethnicity, region, family history, and mutually adjusted for individual risk factors. Estimated PAFs for modifiable risk factors were truncated at a lower limit of 0, as this is the lowest threshold to show a relationship with increased risk. Abbreviations: Abdo obesity, abdominal obesity; Non-HDL-C, non-high-density lipoprotein cholesterol; SEP, socioeconomic and psychosocial risk factors; PAF, population attributable fractions.


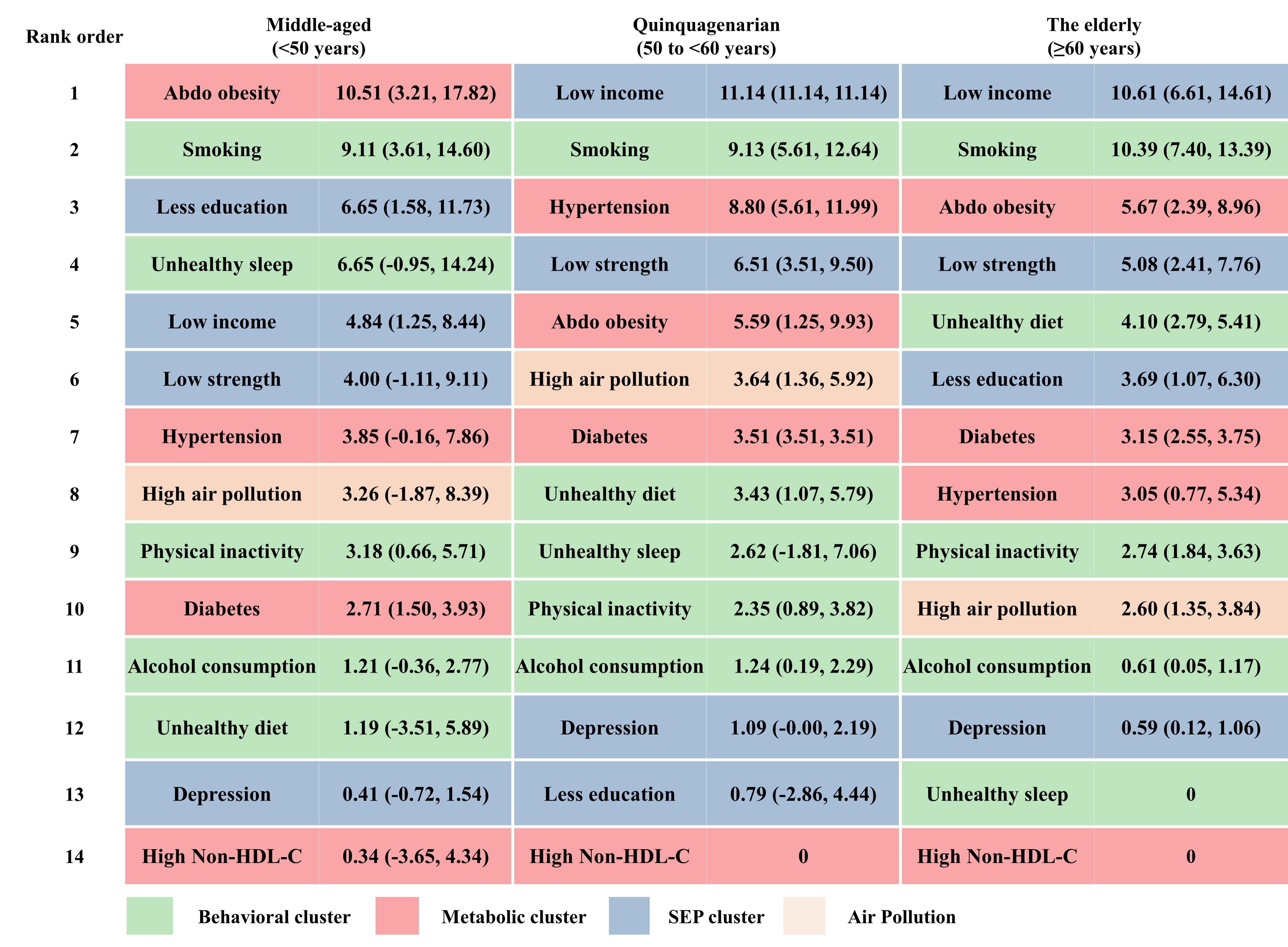
Figure S13. Ranking the population attributable fractions for all-cause mortality associated with the 14 modifiable risk factors among age groups**.**

Models were adjusted for age, sex, ethnicity, region, family history, and mutually adjusted for individual risk factors. Estimated PAFs for modifiable risk factors were truncated at a lower limit of 0, as this is the lowest threshold to show a relationship with increased risk. Abbreviations: Abdo obesity, abdominal obesity; Non-HDL-C, non-high-density lipoprotein cholesterol; SEP, socioeconomic and psychosocial risk factors; PAF, population attributable fractions.
